# Construction of a fusion enzyme for astaxanthin formation and its characterisation in microbial and plant hosts: A new tool for engineering ketocarotenoids

**DOI:** 10.1016/j.ymben.2018.12.006

**Published:** 2019-03

**Authors:** Marilise Nogueira, Eugenia M.A. Enfissi, Ralf Welsch, Peter Beyer, Matias D. Zurbriggen, Paul D. Fraser

**Affiliations:** a School of Biological Sciences, Royal Holloway University of London, Egham hill, Egham TW200EX, United Kingdom; b University of Freiburg, Faculty of Biology II, 79104 Freiburg, Germany; c Faculty of Biology, University of Freiburg and BIOSS Centre for Biological Signalling Studies, University of Freiburg, Schänzlestrasse 18, 79104 Freiburg, Germany

**Keywords:** CrtZ, *Brevundimonas* β-carotene hydroxylase, CrtW, *Brevundimonas* β-carotene ketolase, Astaxanthin, Substrate channeling, Ketocarotenoids, Metabolic engineering, Fusion enzyme

## Abstract

The high-value ketocarotenoid astaxanthin, a natural red colorant with powerful antioxidant activity, is synthesised from β-carotene by a hydroxylase and an oxygenase enzyme, which perform the addition of two hydroxyl and keto moieties, respectively. Several routes of intermediates, depending on the sequence of action of these enzymes, lead to the formation of astaxanthin. In the present study, the enzyme activities of 3, 3ˈ β-carotene hydroxylase (CRTZ) and 4, 4ˈ β-carotene oxygenase (CRTW) have been combined through the creation of “new to nature” enzyme fusions in order to overcome leakage of non-endogenous intermediates and pleotropic effects associated with their high levels in plants. The utility of flexible linker sequences of varying size has been assessed in the construction of pZ-W enzyme fusions. Frist, in vivo color complementation assays in *Escherichia coli* have been used to evaluate the potential of the fusion enzymes. Analysis of the carotenoid pigments present in strains generated indicated that the enzyme fusions only possess both catalytic activities when CRTZ is attached as the N-terminal module. Astaxanthin levels in *E. coli* cells were increased by 1.4-fold when the CRTZ and CRTW enzymes were fused compared to the individual enzymes. Transient expression in *Nicotiana benthamiana* was then performed in order to assess the potential of the fusions in a plant system. The production of valuable ketocarotenoids was achieved using this plant-based transient expression system. This revealed that CRTZ and CRTW, transiently expressed as a fusion, accumulated similar levels of astaxanthin compared to the expression of the individual enzymes whilst being associated with reduced ketocarotenoid intermediate levels (e.g. phoenicoxanthin, canthaxanthin and 3-OH-echinenone) and a reduced rate of leaf senescence after transformation. Therefore, the quality of the plant material producing the ketocarotenoids was enhanced due to a reduction in the stress induced by the accumulation of high levels of heterologous ketocarotenoid intermediates. The size of the linkers appeared to have no effect upon activity. The potential of the approach to production of valuable plant derived products is discussed.

## Highlights

• Metabolons for ketocarotenoid formation.
• The production of valuable chemicals in plants.
• Enzyme fusions to create “new to nature” catalysts.
• The production of ketocarotenoids using transient expression systems.
• Enzyme fusion better suited for plant compared to individual enzymes.

## 1 Introduction

Biosynthetic pathways have evolved to be highly efficient systems that preclude the accumulation of intermediates unless used as precursors by other pathways. The processes of metabolite channeling facilitate the isolation of precursors from their surrounding environment, which enables the localized enrichment of metabolites, to drive high reaction rates and preventing competing pathways accessing common precursors (Winkel, 2004, Zhang, 2011). These advantageous properties are typically achieved through the interaction or co-localization of enzymes and/or their scaffolds in the same cellular vicinity. This can often involve the non-covalent linkage of enzymes, typically in a transient manner. The macromolecular structures that typically facilitate this process have been termed “metabolons” (Srere, 1985). Metabolons have been identified or proposed to operate among a number of pathways involved in specialized/secondary metabolism such as isoprenoids, alkaloids, phenylpropanoids, flavonoids and cyanogenic glucosides (Jorgensen et al., 2005, Winzer et al., 2012) as well as intermediary metabolism such as the tricarboxylic acid cycle (Zhang et al., 2017). The presence of metabolons enabling metabolite channeling has hindered metabolic engineering in both plants and microorganisms. This is due to an inability of the heterologous expressed enzymes to access diffusible precursors because they are already associated with a stoichiometric complex. Alternatively, numerous intermediates accumulate because the heterologous expressed enzyme activities are not coordinated within the microenvironments. As a consequence, intermediates frequently accumulate. Isoprenoid/carotenoid biosynthesis is a key example, with metabolons proposed for isopentenyl diphosphate (IPP) isomerase to phytoene synthase (PSY) (Dogbo et al., 1988, Fraser et al., 2000), and carotene desaturation/isomerisation (Nogueira et al., 2013, Shumskaya et al., 2012).

**Fig. 1:**
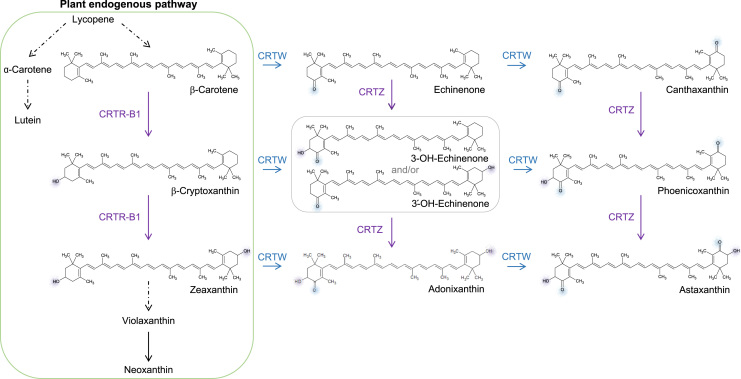
Representative scheme of the ketocarotenoid pathway introduced in plant. Enzyme names are as follow: CRTR-B1, plant carotene β-hydroxylase 1; CRTW, bacterial carotene ketolase and CRTZ, bacterial carotene hydroxylase. The purple and blue shadings depict the position of functional groups (hydroxyl or ketone, respectively). Dashes arrow illustrate more than one enzymatic reactions.

Ketocarotenoids are examples of valuable pigments used across multiple commercial sectors such as the food, feed, health and supplement industries. Total chemical synthesis has been the main route of carotenoid production (Ausich, 1994) using by-products from chemical refining. However, consumer preference is now favoring natural products and the dwindling fossil fuel reserves having a detrimental effect on the environmental credentials of the process. New renewable sources are now urgently required for these molecules, which are only naturally produced by a few microorganisms and plants. These sources are often not amenable to agricultural or fermentation scalability. The advent of metabolic engineering and its evolution into Synthetic Biology has, in the case of ketocarotenoid production, enabled the extension of the pathway from discovery to technical, production and economic feasibility studies in both microorganisms and plant hosts (Misawa and Shimada, 1998, Nogueira et al., 2017). The ketocarotenoids with the greatest potential to act as the most effective colorants include astaxanthin and phoenicoxanthin because of the presence of a keto group in both β-ionone rings (Fig. 1). To form these products from β-carotene a series of hydroxylations and oxygenations (ketolations) must occur (Fig. 1). The best characterized set of genes/enzymes capable of performing this task have been derived from the marine bacteria *Brevundimonas* (Asker, 2017). In this case, a number of intermediates often result at physiological levels of the enzymes (Fig. 1). These intermediates are linked to the fundamental enzymatic parameters of the enzymes, which can deliver multiple routes to the end-product astaxanthin (Fraser et al., 1998; Fig. 1). In marine bacteria the enzymes are termed CRTZ which represents the hydroxylase activity and CRTW which is the oxygenase. Both enzymes are regarded as dioxygenases (Fraser et al., 1997). A mixed-function monooxygenase enzyme capable of forming astaxanthin from β-carotene has been identified in the non-conventional yeast (*Xanthophyllomyces dendrorhous*, formally known as *Phaffia rodozyma*). However, this enzyme requires a specific reductase and endoplasmic reticulum micro-environment (Chi et al., 2015).

In the present study, the potential to create an active fusion protein between the CRTZ and CRTW enzymes has been explored using flexible linkers (Chen et al., 2013, Siu et al., 2015). The aim was to augment efficient transfer of products/precursors through the creation of a closer microenvironment. To characterize the potential outputs from the fusion protein variants, color complementation in *Escherichia coli* has been used as well as transient expression in *Nicotiana benthamiana.*

## 2 Materials and methods

### 2.1 Plasmid construction

#### 2.1.1 Plasmids for bacterial expression

**Fig. 2:**
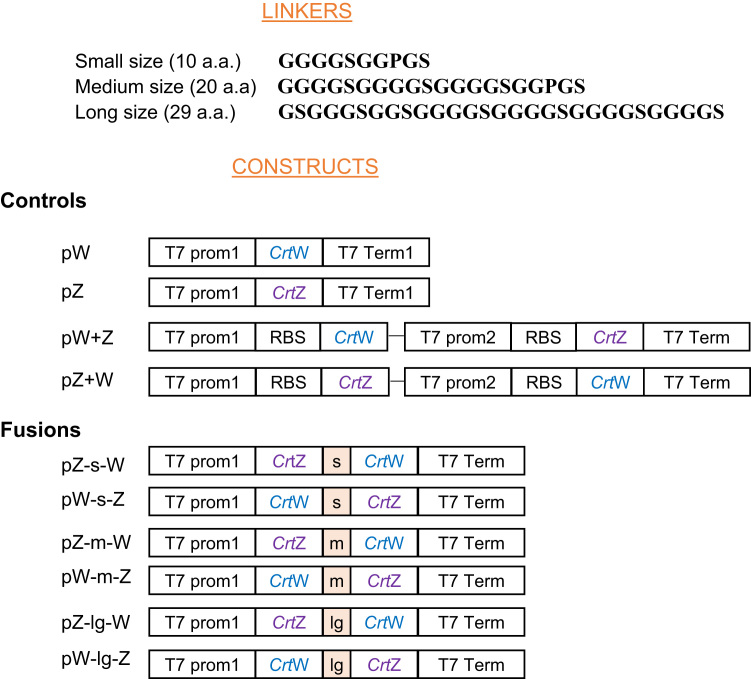
Linker sequences and structures of the different *Crt*W & *Crt*Z constructs for expression in *E.coli*. G, glycine; S, serine; P, proline; a.a., amino acid; Prom, promoter; RBS, Ribosome Binding Site; Term, terminator; s, small linker; m, medium linker; lg, long linker. The vector used to create the constructs was a pETDuet^TM^-1 vector. The nucleotide sequences of the linkers are given in Supplementary Table 1.

Several plasmids were created in order to test the CRTZ and CRTW fusion enzymes: pZ-s-W, pZ-m-W, pZ-lg-W, pW-s-Z, pW-m-Z, pW-lg-Z, with different size linkers, small (s), medium (m) and long (lg) (Fig. 2). The nucleotide sequences of the linkers are described in Supplementary Table 1. Other plasmids, which were used as controls were also constructed: pZ, pW, pZ+W, pW+Z. The pETDuet™-1 vector (pET designated as p-Ø), which allows the coexpression of two target genes was used as a backbone to build the different plasmids. Based on gel analysis, the copy number of the pETDuet™-1 plasmid is around 40 copies per cell (Duet™ vectors, User protocol TB340, NOVAGEN). The plant pZ+W vector (Supplementary Fig. 1), which harbors the plant codon-optimized 3, 3ˈ β-carotene hydroxylase (*Crt*Z) and 4, 4' β-carotene ketolase (*Crt*W) genes from the marine bacterium *Brevundimonas* sp strain SD212, was used as a template to clone the *Crt*Z and *Crt*W genes. *Crt*Z and *Crt*W primers were designed with a 36 nt flanking homologous regions to the adjacent pET backbone or to parts of the linkers to be inserted, as described in Beyer et al. (2015). Each pair of primers was consequently unique to each construct (Supplementary Table 1). PCR products obtained using the specific primers and the plant pZ+W vector were gel extracted. When the construct contained only one transcriptional unit, it was inserted into the first multiple cloning site (MCS) of the pET vector. In the case of pZ+W and pW+Z vectors, which harbor two transcriptional units, both multiple cloning sites were used. The pET vector was cut with NcoI and BamHI or NdeI and PacI restriction enzymes when utilized for its first or second MCS, respectively. The fragment of interest was subsequently gel extracted. Gibson assembly (Gibson et al., 2009) with the appropriate purified PCR products and purified cut pET backbone was performed in order to build each plasmid. The sequences of the newly made plasmids were verified by sequencing.

#### 2.1.2 Plasmids for plant expression

The plant pZ+W vector, described in Section 2.1.1, was made from the pZK3B binary vector (Kuroda et al., 2010). It was modified to create the plant pZ-s-W, pZ-m-W and pZ-lg-W vectors by removing a section between *Crt*Z and *Crt*W in pZ+W (Supplementary Fig. 1), including a region of *Crt*Z and *Crt*W sequences and replacing them with the corresponding Z-W (linked) sequences from the bacterial constructs. The pZ+W vector was cut with the *Bsp*HI restriction enzyme, once in *Crt*Z and once in *Crt*W gene. Subsequently, the fragment of interest was gel extracted. PCR products were obtained using the bacterial pZ-s-W, pZ-m-W and pZ-lg-W with specific primers (Supplementary Table 1). Gibson assembly was then performed to obtain the plant pZ-s-W, pZ-m-W and pZ-lg-W vectors (Supplementary Fig. 1). The sequences of the newly made plasmids were verified by sequencing.

### 2.2 Biological materials and growth conditions

*Escherichia coli* BL21-CodonPlus-RIL were grown in Luria-Bertani (LB) broth supplemented with the appropriate antibiotics. A preculture (4 mL) was inoculated from a single colony and grown overnight, shaking at 37 °C, in selective medium. An aliquot of the preculture (1 mL) was then added to fresh selective medium (50 mL) and grown first for 2 h at 37 °C and then at 28 °C for different lengths of time (1.5 h, 3 h, 6 h, 9 h, 22 h and 26 h for the time series, 20 h otherwise). Level of repetition was n = 3 unless stated otherwise. *Nicotiana benthamiana* plants were greenhouse grown (25 °C day/15 °C night), with supplementary lighting (16 h light/8 h dark) in pots containing M3 professional growing medium (Scotts Levington®, UK).

### 2.3 Transformations

*E. coli* competent cells were transformed following the heat shock method. *E. coli* competent cells (25 µL) were thawed on ice. Plasmid DNA (~ 100 ng) was mixed gently with the cells. The mixture was incubated on ice for 30 min and then transferred into a water bath at 42 °C for 45 s followed by incubation on ice for 2 min. SOC media (Super Optimal broth with Catabolite repression; 250 µL) was added. The mixture was incubated at 37 °C for 1 h, gently shaking at 180 rpm. Cells were plated on fresh LB-agar plates, supplemented with appropriate antibiotic selection and incubated overnight at 37 °C.

*N. benthamiana* leaves were transformed using *Agrobacterium tumefaciens* C58C1-mediated transient transformation. The detailed protocol is described in Sparkes et al. (2006). Overnight cultures (5 mL) of *Agrobacterium* harboring the plasmid of interest were transferred into 50 mL of induction media and grown again overnight at 28 °C, shaking at 220 rpm. Overnight cultures (50 mL) were then centrifuged for 10 min at 2200 g in an Eppendorf centrifuge 5810 R. The supernatants were discarded and the pellets were resuspended in induction medium (5 mL). The centrifugation step was repeated and the pellets were resuspended in infiltration medium to an OD_600_ of 0.5. Each culture of *Agrobacterium* carrying a plasmid of interest was combined in an equal volume to a culture of an *Agrobacterium* harboring the p19 vector to supress gene silencing (Voinnet et al., 2003). A 1 mL syringe was used to infiltrate the *Agrobacterium* culture mixture on the underside of the leaf. Several infiltrations of the same culture mixture were performed on the same leaf until most of the leaf surface was agro-infiltrated. Successful infiltration was indicated by a temporary change of in leaf color. Four to five leaves from three plants were agro-infiltrated per construct. Leaves were collected and frozen in liquid nitrogen at various days after infiltration depending on the experiment. The three leaves with the best colored phenotype were then pooled for each plant.

### 2.4 Carotenoid analysis

Carotenoids produced in *E.coli* were extracted from culture media. Bacterial culture (~ 50 mL) was centrifuged for 10 min at 2200 g in a 5810 R centrifuge (Eppendorf). The pellet was resuspended in 2.5 mL of LB medium, of which 2 mL was placed in two fresh Eppendorf tubes (1 mL/tube). Tubes were centrifuged for 5 min at 4600 g in a Heraeus Pico 21 centrifuge (Thermo Scientific). In the case of the time series experiment, 10 mL of culture split in two tubes were pelleted per biological repetition for each time point. Carotenoids were extracted from the pellet by the addition of one volume of acetone (300 µL) twice. Tocopherol acetate (1 µg) was added as an internal standard with the first volume of acetone. Tubes were centrifuged for 5 min at 4600 g for each extraction. Subsequently, one equal volume of petroleum ether/diethyl ether (2:1; v/v) and water (700 µL) were added. Samples were centrifuged at 20,000 g for 3 min. The organic phase, containing the carotenoids, was placed in a fresh Eppendorf tube and the aqueous phase was re-extracted with an equal volume of petroleum ether: diethyl ether (2:1; v/v). Samples were centrifuged at 20,000 g for 3 min. Organic phases were pooled and dried using the Genevac EZ.27 (SP scientific). Dried samples were stored at −20 °C and resuspended in ethyl acetate or ethyl acetate/acetonitrile (1:4; v/v) prior to spectrophotometric and chromatographic analysis. Carotenoids produced in *N. benthamiana* leaves were extracted in three technical replicates from 15 mg of freeze-dried leaves. The extraction of carotenoids from plant tissue, the Ultra High Performance Liquid Chromatography with photo diode array detection (UPLC-PDA) analysis, and quantification of carotenoids are described in detail in Nogueira et al. (2013). The identity of (keto) carotenoids was confirmed by co-chromatography with authentic standards on multiple chromatographic systems.

### 2.5 mRNA expression study

RNA was extracted from 100 mg of transiently transformed fresh *N. benthamiana* leaves with the RNeasy mini kit (QIAGEN) following the manufacturer instructions. The extraction was performed on three plants for each vector transformation. Synthesis of cDNA was performed from 500 ng of RNA using the QuantiTect Reverse Transcription Kit from QIAGEN following the manufacturer instructions. PCR was then carried out with illustra^TM^ puReTaq Ready-To-Go PCR Beads from GE Healthcare, using 2 µL of cDNA previously synthesised, the specific primers at 7.5 pmol and water in 25 µL total volume. A standard three-step endpoint PCR cycling protocol was used, consisting of an initial denaturation at 95 °C then 25 cycles of denaturation at 95 °C for 30 s, annealing at 55 °C for 30 s and elongation at 72 °C for 75 s, with a final extension at 72 °C for 5 min.

### 2.6 Statistics

IBM SPSS Statistics 21 software was used to determine the significant differences between different groups. T-tests and one way ANOVA were used to compare one independent variable that consisted of two independent groups or more than two independent groups, respectively. Repeated measures ANOVA test was used to compare one independent variable that consisted of more than two dependent groups. For the one way ANOVA, if homogeneity of variance (Levene test) was assumed, the Tukey post Hoc test was performed, if it was violated, the Games-Howell post Hoc test was used. P-values were calculated and represented as follow: *P* < 0.05, *P* < 0.01, and *P* < 0.001 were indicated by *, **, and ***, respectively, when appropriate. Supplementary Table 2 describes the statistical tests performed in this paper and all the p-values obtained with the SPSS software.

## 3 Results

### 3.1 Construction of new fusion enzymes for ketocarotenoid production

#### 3.1.1 Generation of fused CrtZ and CrtW genes using linkers of different sizes

The plant codon-optimized 3, 3ˈ β-carotene hydroxylase (*Crt*Z) and 4, 4ˈ β-carotene ketolase (*Crt*W) genes from the marine bacterium *Brevundimonas* sp strain SD212 were chosen to create the new fusion enzymes as they have previously been shown to be efficient at producing ketocarotenoids in plants when expressed individually (Mortimer et al., 2016, Mortimer et al., 2017, Nogueira et al., 2017). The two proteins were expressed as translational fusions, separated by linker spacers of three different sizes (small, medium and long; Fig. 2). Flexible linkers, rich in small or hydrophilic amino acids, were utilized to keep the two CRT enzymes in close vicinity and simultaneously allowing interaction between domains (Fig. 2). Several constructs were made to evaluate the different possible combinations of the fusion enzymes (Fig. 2).

#### 3.1.2 Functional screening of fused ketocarotenogenic enzymes in Escherichia coli

**Fig. 3:**
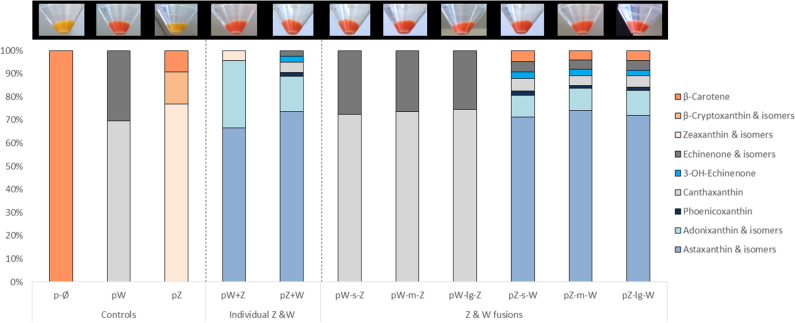
Assessment of carotenoid production by the different fusion constructs in *E.coli*. β-carotene *E. coli* producer*,* containing pACCAR16ΔcrtX, were transformed with the vector of interest and the empty vector (p-Ø). The pictures show bacteria pellets at the end of the culture. The yellow hue corresponds to the presence of the following carotenoids, β-carotene, β-crypthoxanthin and zeaxanthin, while the red color is due to ketocarotenoids. The graph represents the carotenoid composition of the bacteria as a percentage of total carotenoids. W, *Crt*W; Z, *Crt*Z; s, small linker; m, medium linker; lg, long linker.

**Fig. 4:**
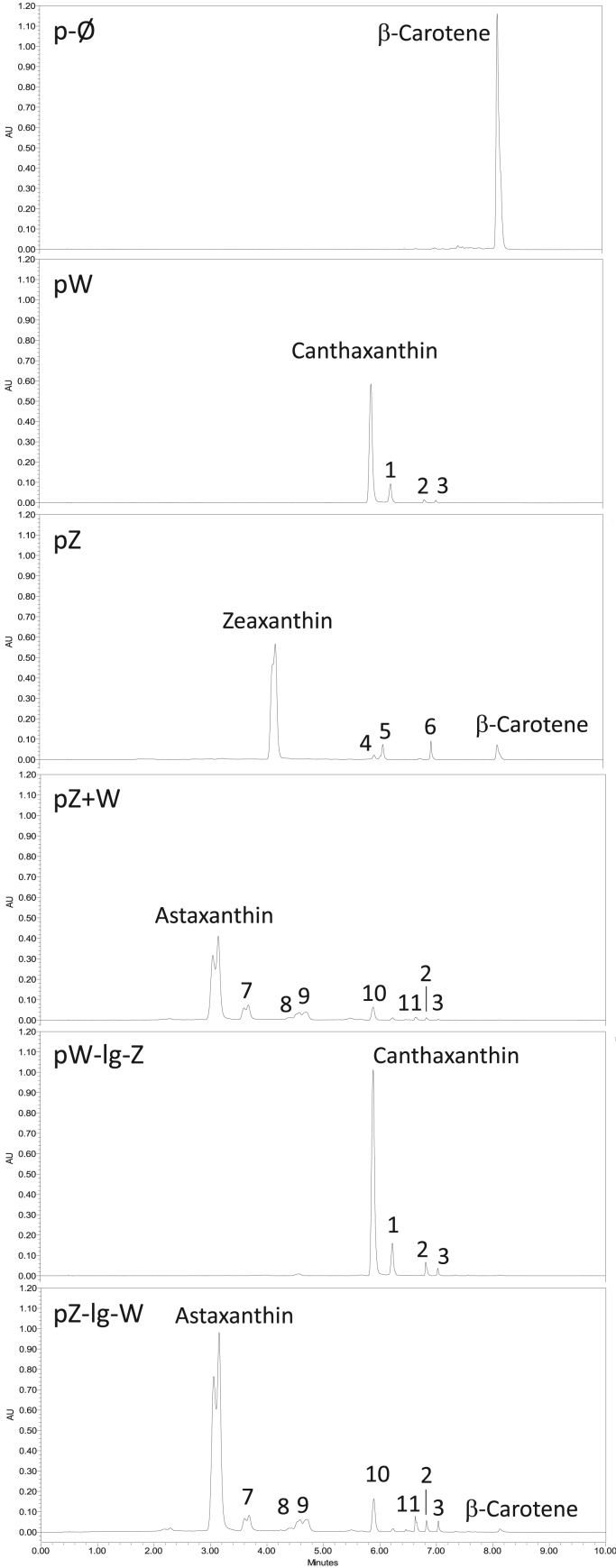
Chromatographic carotenoid profiles of the *E.coli* fusions and controls. Each time, *E. coli* was simultaneously transformed with the vector of interest and pACCAR16ΔcrtX (β-carotene producer). The chromatographic carotenoid profiles were obtained by UPLC and recorded at 470 nm. Profiles for the fusion with different size linkers were very similar so only the profiles of the fusions linked with the long linker are presented. Similarly, only pZ+W (individual) is shown. 1, putative echinenone isomer 1; 2, putative echinenone isomer 2; 3, echinenone; 4, putative β-cryptoxanthin isomer 1; 5, putative β-cryptoxanthin isomer 2; 6, β-cryptoxanthin; 7, adonixanthin; 8, phoenicoxanthin; 9, putative adonixanthin isomer; 10, canthaxanthin; 11, 3-OH-echinenone.

In order to assess the ability of the different fusion enzymes in producing ketocarotenoids, the fusions as well as the control constructs were expressed in an *E. coli* host. Previously, *E.coli* bacteria were transformed with the pACCAR16ΔcrtX vector (Misawa et al., 1995), carrying the *Pantoea ananatis* carotenogenic genes *Crt*E, *Crt*B, *Crt*I and *Crt*Y, which are responsible for the conversion of isopentenyl pyrophosphate/dimethylallyl pyrophosphate to β-carotene, the precursor of CRTZ and CRTW enzymes (Fig. 1). Colored bacteria were obtained, revealing the presence of carotenoids (yellow) or ketocarotenoids (red) (Fig. 3). The main (keto) carotenoid produced for each construct was easily identifiable in each corresponding chromatogram (Fig. 4) and were as follows: β-carotene for p-Ø, the empty vector; zeaxanthin for pZ, canthaxanthin for p-W, pW-s-Z, pW-m-Z and pW-lg-Z; astaxanthin for pW+Z, pZ+W, pZ-s-W, pZ-m-W and pZ-lg-W. The detailed carotenoid composition of those bacteria is presented in Fig. 3 and contents are displayed in Supplementary Table 3. Bacteria expressing the pW-Z fusions did not accumulate astaxanthin such as bacteria producing the individual CRTZ and CRTW enzymes (pW+Z & pZ+W), but instead had a similar carotenoid profile to the *E.coli* expressing the pW construct. The CRTZ enzyme seemed to not be functional when positioned at the C-terminus of the fusion enzyme as in the pW-Z fusion constructs, independent of the linker length. On the contrary, the Z-W fusion enzymes were functional as the corresponding transformed *E.coli* had similar carotenoid profiles to the individual enzymes and both accumulated the final product of the pathway: astaxanthin. The size of the linkers (small, medium and long) used in the Z-W fusion constructs did not seem to have an impact on the carotenoid content produced by the respective bacteria (Supplementary Table 3).

### 3.2 Ketocarotenoid production in E.coli using enzyme fusions

Further analyses were conducted to compare the carotenoid content in *E.coli* expressing pZ+W and pZ-m-W over six time points (1.5 h, 3 h, 6 h, 9 h, 22 h and 26 h after protein induction at 28 °C). Carotenoid content in µg/gDCW is described in Table 1 and in mg/L in Supplementary Table 4. The chromatographic carotenoid profiles of the *E.coli* studied over time are presented in Supplementary Fig. 1. The precursor β-carotene, the final product astaxanthin as well as all the ketocarotenoid intermediates (echinenone, 3-OH-echinenone, adonixanthin, canthaxanthin and phoenicoxanthin) were already present at the first time point (1.5 h) and then were continuously produced throughout the cultivation. From the 9 h time point, the astaxanthin content was significantly greater (up to 1.4-fold) with the pZ-W fusion strain compared to pZ+W, while the levels of adonixanthin, a direct precursor of astaxanthin, were significantly lower (2–3-fold) in pZ-W compared to that of the pZ+W strain (Table 1). A reduction of the total ketocarotenoid intermediates was also observable in pZ-W (1.2-fold). The fused Z-W enzyme was more efficient at producing astaxanthin compared to the individual enzymes. This seems to be due to a faster conversion of adonixanthin into astaxanthin by the fused Z-W enzyme consequently leading to lower levels of adonixanthin in the pZ-W strain compared to that in pZ+W *E.coli*.

**Table 1:** Carotenoid content (µg/gDCW) in pZ+W (individual) and pZ-W (fusion) *E.coli* over six time points. *E. coli* was simultaneously transformed with the vector of interest and the pACCAR16ΔcrtX (β-carotene producer). Bacterial precultures were grown at 37 °C and subsequently incubated at 28 °C for 1.5 h, 3 h, 6 h, 9 h, 22 h and 26 h. Carotenoid levels are represented as µg/gDCW, n = 3. The mean data are shown as ± SD. Values in bold indicate where significant differences have been found when comparing pZ-W to pZ+W.

		1.5 h	3 h	6 h	9 h	22 h	26 h
Astaxanthin	pZ+W	76.3 ± 8.4	75.6 ± 6.8	207.0 ± 31.4	332.4 ± 37.2	440.8 ± 24.1	457.2 ± 50.5
	pZ-W	93.4 ± 5.9*	83.3 ± 8.7	189.9 ± 22.0	441.8 ± 25.0*	610.4 ± 11.2***	576.4 ± 30.4*
Adonixanthin	pZ+W	38.8 ± 6.2	24.6 ± 10.4	47.6 ± 2.4	89.1 ± 8.2	68.2 ± 3.9	64.4 ± 8.4
	pZ-W	32.1 ± 2.4	17.3 ± 4.3	15.6 ± 2.8***	32.8 ± 10.1**	36.2 ± 2.9***	29.6 ± 2.5**
Phoenicoxanthin	pZ+W	45.1 ± 3.0	23.5 ± 0.3	22.1 ± 2.5	26.5 ± 3.2	24.1 ± 3.2	23.9 ± 4.5
	pZ-W	52.2 ± 4.5	27.1 ± 0.6***	26.9 ± 5.1	25.0 ± 2.2	22.4 ± 0.3	20.2 ± 1.5
Canthaxanthin	pZ+W	72.0 ± 12.6	44.7 ± 6.1	48.5 ± 13.6	78.7 ± 26.3	66.3 ± 21.7	64.8 ± 25.0
	pZ-W	92.0 ± 16.4	59.6 ± 7.3	86.0 ± 30.7	74.4 ± 11.7	63.1 ± 1.2	55.2 ± 6.1
3-OH-Echinenone	pZ+W	100.7 ± 0.9	48.4 ± 6.8	64.9 ± 6.0	92.2 ± 6.8	51.2 ± 6.6	49.0 ± 8.0
	pZ-W	109.6 ± 2.5**	48.4 ± 5.4	46.4 ± 4.0*	84.4 ± 3.3	57.2 ± 6.1	48.8 ± 2.6
Echinenone & isomer	pZ+W	199.5 ± 5.6	95.0 ± 5.8	89.9 ± 10.5	97.6 ± 12.1	68.7 ± 4.9	67.3 ± 5.2
	pZ-W	221.7 ± 10.5*	99.1 ± 6.1	88.5 ± 11.5	92.8 ± 5.1	73.3 ± 2.8	67.7 ± 1.7
β-Carotene	pZ+W	250.0 ± 6.8	112.5 ± 8.4	106.2 ± 6.0	121.9 ± 1.3	111.6 ± 14.3	109.7 ± 10.8
	pZ-W	286.0 ± 18.0*	126.1 ± 4.8	105.9 ± 4.9	144.1 ± 16.8	166.4 ± 4.5**	157.2 ± 11.0**
Total ketocarotenoid	pZ+W	532.2 ± 22.9	311.9 ± 23.5	480.1 ± 60.0	716.5 ± 88.3	719.2 ± 48.7	726.6 ± 92.7
	pZ-W	601.0 ± 34.4*	334.9 ± 16.8	453.3 ± 22.5	751.2 ± 46.9	862.6 ± 16.5**	797.9 ± 38.9
Total	pZ+W	782.3 ± 22.9	424.4 ± 23.5	586.2 ± 60.0	838.4 ± 88.3	830.8 ± 48.7	836.3 ± 92.7
	pZ-W	887.0 ± 52.3*	461.0 ± 21.6	559.2 ± 27.0	895.3 ± 63.0	1029.0 ± 14.6***	955.0 ± 49.8

P < 0.05, P < 0.01 and P < 0.001 are designated by *,**, and ***, respectively. Computed p**-**values are tabulated in Supplementary Table 2, as well as the p-values corresponding to the difference between time points for each carotenoid.

### 3.3 Transient production of ketocarotenoids in N. benthamiana using enzyme fusions

**Fig. 5:**
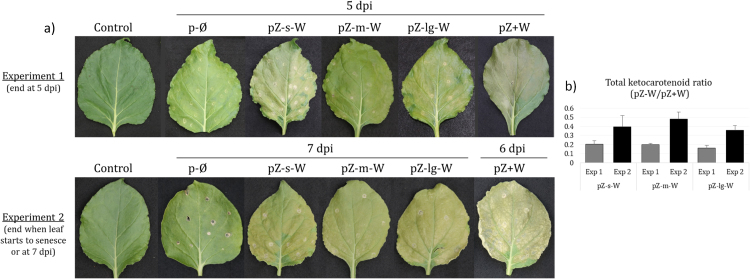
*N. benthamiana* leaf phenotype at different days after agro-infiltration with the fusion and control constructs. a) leaf phenotype at the end of the experiments 1 & 2. b) Ratio of total ketocarotenoids calculated by dividing the total ketocarotenoid content of pZ-W (fusion) by that of the pZ+W (individual). Control, non agro-infiltrated; p-Ø, empty vector; pZ-x-W, fusion enzymes with different size linker (x = small, medium or long); pZ+W, individual enzymes.

The fused *Crt*Z-*Crt*W genes (with the three different size linkers) were inserted in a binary vector harboring the RuBisCo small subunit transit peptide from pea and under the control of the *35 S* promoter. The control vector had both *Crt*Z and *Crt*W under the control of *35 S* promoter as independent transcriptional units (Supplementary Fig. 2). Agro-infiltration experiments of *N. benthamiana* leaves were performed. The presence of the mRNA encoding the fusion enzymes in the pZ-W agro-infiltrated leaves has been demonstrated by RT-PCR (Supplementary Fig. 3). The mRNAs corresponding to parts of *Crt*Z and *Crt*W genes were amplified from pZ+W and pZ-W (pZ-s-W, pZ-m-W and pZ-lg-W) leaves but not from the control leaves transformed with the empty vector (p-Ø), whilst the mRNAs transcribed from the linker region were only obtained from the fusion pZ-W leaves. The tobacco leaves agro-infiltrated with pZ+W and the pZ-W vectors showed a yellow-brown hue, and generally, the pZ+W agro-infiltrated leaves had a darker color (Fig. 5). This phenotype corresponded to the accumulation of ketocarotenoids in the agro-infiltrated leaves (Table 2, Supplementary Fig. 4 and Supplementary Table 5).

**Table 2:** Carotenoid content in *N. benthamiana* agro-infiltrated leaves collected at the onset of leaf senescence or at 7 dpi (experiment 2). Carotenoid levels are given in μg/g dry weight. Three plants were agro-infiltrated with each vector. Three leaves were pooled per plant and three technical replicates were analysed. The mean data are shown as ± SD. Data with significant differences when comparing pZ+W (individual) and pZ-W (fusion) to the empty vector (p-Ø) for the non ketocarotenoids and comparing pZ-W to pZ+W for the ketocarotenoids are shown in bold.

		p-Ø	pZ+W	pZ-s-W	pZ-m-W	pZ-lg-W
Caro.	Neoxanthin	412.3 ± 45.6	312.2 ± 57.0	360.3 ± 12.0	333.6 ± 21.5	283.0 ± 26.4
	Violaxanthin	326.3 ± 35.0	195.3 ± 14.9**	244.7 ± 38.4	227.4 ± 38.3*	219.8 ± 9.2*
	Zeaxanthin	380.4 ± 94.3	348.7 ± 38.1	402.1 ± 7.9	429.4 ± 70.4	355.2 ± 28.2
	Lutein	1096.5 ± 143.8	797.4 ± 192.8	1009.7 ± 80.8	880.3 ± 91.7	744.3 ± 65.6
	β-carotene	423.6 ± 54.6	103.9 ± 18.6***	227.6 ± 43.9***	185.1 ± 10.8***	153.1 ± 21.2***
	Total carotenoid	2639.1 ± 310.5	1757.5 ± 318.6**	2244.5 ± 105.9	2055.8 ± 163.6	1755.4 ± 140.9**
Keto.	Astaxanthin	nd	14.7 ± 3.0	6.0 ± 6.2	8.6 ± 2.5	3.6 ± 2.1*
	Adonixanthin	nd	19.5 ± 1.8	12.3 ± 4.6	19.4 ± 4.7	12.5 ± 1.7*
	Phoenicoxanthin	nd	32.2 ± 7.9	6.9 ± 4.6***	9.5 ± 0.9***	6.6 ± 2.0***
	Canthaxanthin	nd	162.3 ± 22.4	7.0 ± 3.1***	6.7 ± 4.2***	2.9 ± 2.0***
	3´-OH-Echinenone	nd	28.0 ± 1.9	17.0 ± 1.1*	16.5 ± 0.4*	16.2 ± 0.5*
	3´-OH-Ech. isomer	nd	43.2 ± 11.6	56.9 ± 21.1	78.2 ± 13.0	58.1 ± 10.4
	Echinenone	nd	44.5 ± 15.1	29.8 ± 1.6	26.3 ± 2.9	23.2 ± 3.4
	Total ketocarotenoid	nd	344.3 ± 44.9	135.9 ± 37.8***	165.2 ± 24.5***	123.0 ± 15.7***
	Total	2639.1 ± 310.5	2101.8 ± 359.5	2380.5 ± 95.7	2221.0 ± 184.7	1878.4 ± 146.8*

*,*P* < 0.05; **, *P* < 0.01 and * ** , *P* < 0.001. Nd, not detected; Caro., carotenoids; Keto., ketocarotenoids. Computed p-values are tabulated in Supplementary Table 2.

Two independent agro-infiltration experiments were conducted. The first experiment was designed to compare the ketocarotenoid levels produced by the fused (Z-W) or individual (Z+W) enzymes over a defined period of time, while the second experiment was set up to allow the greatest accumulation of ketocarotenoids in each agro-infiltrated leaf. The first experiment was stopped at 5 day post infiltration (dpi), which corresponded to the senescing of the first pZ+W leaves. All agro-infiltrated leaves were then collected (Supplementary Table 5). Importantly, at 5 dpi the control and fusion pZ-W leaves were not showing any signs of senescence. In the second experiment, each agro-infiltrated leaf was independently harvested when it showed signs of senescence until 7 dpi when all remaining leaves were harvested (Fig. 5). The details of the harvest of the leaves of this experiment are presented in Supplementary Fig. 5. Remarkably, the majority of pZ+W tobacco leaves were harvested before the fusion pZ-W leaves, implying that ketocarotenoid formation mediated by the fusion enzyme causes less stress and therefore delayed senescence compared to individually expressed CrtZ and CrtW. The ratio of total ketocarotenoids of pZ-W/pZ+W increased in the prolonged experiment compared to the 5 dpi one (Fig. 5). The total ketocarotenoid content of the pZ-W leaves increased by up to almost 2-fold in the prolonged experiment compared to the 5 dpi one and the levels of astaxanthin in pZ-s-W and pZ-m-W leaves were no longer statistically different compared to the level in pZ+W leaves (Table 2). This highlights the potential for the pZ-W leaves to maintain ketocarotenoid production and accumulation over a longer duration, whereas the pZ+W leaves seemed to be at their maximum capacities. In both experiments, the pZ+W tobacco leaves had significantly greater quantities of total ketocarotenoids compared to the pZ-W leaves (Table 2, Supplementary Table 5). However, for pZ+W leaves, no increase of total ketocarotenoids nor astaxanthin was observed during the prolonged experiment compared to the 5 dpi one. The significant difference in total ketocarotenoids between pZ+W and pZ-W leaves in the prolonged experiment was due to the significantly higher content of some of the intermediates in pZ+W leaves (phoenicoxanthin, canthaxanthin and 3´-OH-echinenone; Table 2).

In order to compare the carotenoid composition of the pZ+W and pZ-W agro-infiltrated leaves, the best plant producer of ketocarotenoids were selected. Thus, this analysis reflects the maximum potential obtained for each vector tested. The carotenoid composition of the best agro-infiltrated plants is displayed in Supplementary Fig. 6. On a percentage basis, the astaxanthin produced by the fusion enzymes was similar to the one produced by the individual enzymes, especially for pZ+W and pZ-s-W (4.1% and 3.9%, respectively). The percentage of ketocarotenoid intermediates in the fusion pZ-W leaves contributed to 48% of the total ketocarotenoids and β-carotene content and was 1.6-fold lower than the percentage in pZ+W leaves. The fusion enzymes managed to generate similar levels of astaxanthin in the leaves without accumulating high levels of intermediates and without decreasing the carotenoid endogenous pool too drastically, which resulted in better leaf vigour and a higher potential for ketocarotenoid production improvement. There was no significant effect of the size of the linkers on the carotenoid levels produced during the agro-infiltration experiments when comparing pZ-s-W, pZ-m-W and pZ-lg-W in *N. benthamiana*.

## 4 Discussion

Fusion enzymes have been successfully used in the past to channel intermediates towards a specific product, for instance for alpha-farnesene or glycerol production (Salles et al., 2007, Wang et al., 2011, Zhang, 2011). Moreover, fusion enzymes provide benefits such as alleviating the need for two promoters, since the fused genes are part of only one transcriptional unit, and concurrently reducing the size of the construct. In addition, the elimination of multiple promoters and combined open reading frames reduces homology and thus the potential of gene silencing.

In this study, functional fusion ketocarotenogenic enzymes (Z-W) have been created and evaluated against the two individual enzymes (Z + W) in *E.coli* and *N. benthamiana*. In both hosts, the pZ-W fusion enzymes allowed the production of the end-product astaxanthin to statistically higher or similar levels compared to that of pZ+W while reducing the amount of intermediates, with the exception of pZ-lg-W in *N. benthamiana*, which contained significantly less astaxanthin (Table 1, Supplementary Table 4, Table 2 and Supplementary Fig. 6). In *N. benthamiana*, the greatest difference in terms of intermediates was the accumulation of canthaxanthin in pZ+W leaves (Supplementary Fig. 6). Canthaxanthin represented 40% of the total ketocarotenoids and β-carotene in pZ+W and only around 3% in pZ-W leaves. In terms of quantity, canthaxanthin amounts were 56-fold greater in pZ+W than in pZ-W leaves (Table 2). All other levels of intermediates only varied up to 5-fold. Canthaxanthin is synthesised from β-carotene in two enzymatic steps requiring only CRTW (Fig. 1). This quantitative change of intermediate composition between pZ+W and pZ-W leaves could indicate that canthaxanthin is channeled more efficiently in tobacco when the hydroxylase (CRTZ) is fused to CRTW. Furthermore, the main intermediate in pZ-W leaves was the 3ˈOH-echinenone isomer (Table 1). Its synthesis requires the participation of both enzymes. This also highlights the enhancement of the enzymes interaction when fused together. The positive effect of producing less non-endogenous intermediate ketocarotenoids in *N. benthamiana* leaves was to reduce the plant's stress and consequently extend the life of the agro-infiltrated leaves (Table 2, Supplementary Fig. 5).

The level of astaxanthin was significantly different between pZ-W and pZ+W in *N. benthamiana* when agro-infiltrated for 5 days but then no longer at 7 days (Table 2 and Supplementary Table 6). Although, the fusion enzymes seemed slower to start with, when given enough time, in most cases, the pZ-W transformed host accumulated a similar or greater level of astaxanthin compared to the one transformed with the individual enzymes. This suggests that potentially greater quantities of astaxanthin could be obtained in pZ-W, for instance if stably transformed in a plant host. The expected, self-evident, effects of metabolite or substrate channeling by enhanced local proximity are a reduction of the intermediate leakage and the acceleration of the overall reaction rates. However, as it was found here, in planta, and as it was shown in Poshyvailo et al. (2017) using a simple model supported by stochastic simulations, the acceleration of the reaction is not always happening. In the case of an enzyme-enzyme fusion, it is likely that the enzymatic activities are slightly impaired by the fusion. A positive outcome of a slower rate of synthesis could be linked to the plant host having more time to adapt and to manage the non-endogenous metabolites produced. A reduced level of ketocarotenoid intermediates, their slower accumulation in the plant as well as the maintenance of the endogenous carotenoid levels could explain the delayed senescing of the pZ-W fusion leaves compared to the pZ+W leaves. The slower formation could also reflect changes in the allosteric regulation of the enzyme(s) activity directly. Less intermediates accumulated but the higher proportion of astaxanthin could have contributed to feedback inhibition and thus the slower rate of formation.

Antibodies were not available to test the integrity of the fusion enzymes. However, the chimeric mRNAs were transcribed as expected (Supplementary Fig. 3), even for the W-Z fusion which was non-functional (Fig. 3). The CRTZ enzyme was only operational when linked with the N-terminal of CRTW in the Z-W fusion enzymes, while the CRTW enzyme was functional in both conformations (Fig. 3). Furthermore, the metabolite profiles of the Z-W fusions were different from that of the individual enzymes (Z+W). Altogether, this suggests that the integrity of the fusion enzymes was kept. This might be explained by the structures of the enzymes. Prediction of the 3D structures of the enzymes were obtained with high confidence (99% and 81% of the residues were modelled at > 90% of confidence for CRTW and CRTZ, respectively; Supplementary Fig. 7). The CRTW and CRTZ models are illustrated in Supplementary Fig. 8. Their respective predicted active site as well as the topology of the transmembrane helices are also depicted. It is possible that the fusion of the enzymes has resulted in a slight change in their conformation near the linker region. In CRTZ, the first helix is very close to the N-terminal (12 amino acid) and this helix is predicted to be involved in the active site. In CRTW, the first helix is also close to the N-terminal (18 a.a.) but the helix is not predicted to take part in the active site formation. Therefore, when the CRTZ is fused by its N-terminal, the impact on its active site shape could likely be more severe than in CRTW, which could potentially prevent the CRTZ enzyme from functioning.

Three different size linkers (10, 20 and 29 a.a.) were used to allow different levels of flexibility between the two fused enzymes and potentially enhance their interaction (Fig. 1). Statistically, there was no difference in terms of ketocarotenoid production between the three fusion enzymes (pZ-s-W, pZ-m-W and pZ-lg-W) when expressed in *E.coli* or *N. benthamiana*. However, the level of astaxanthin of pZ-lg-W in *N. benthamiana* was significantly lower compared to the control, while those for pZ-m-W and pZ-s-W were not (Table 2). Therefore, shorter linkers probably enabled closer interaction of the enzymes and enhanced the fusion enzymes capacity at channeling intermediates.

The carotenoid and ketocarotenoid profiles of the fusion and individual enzymes obtained in *E.coli* and *N. benthamiana*, although qualitatively similar, were quantitatively different. CRTZ and CRTW enzymes did not produce astaxanthin with the same efficiency in both hosts. In *E.coli*, astaxanthin accumulated up to almost 60% of the total ketocarotenoid and β-carotene but only to 4% in *N. benthamiana*, while β-carotene represented up to 15% and 50%, respectively (Table 1 and Supplementary Fig. 6). The ketocarotenogenic enzymes were more efficient at converting β-carotene into astaxanthin in the bacterial host compared to the plant one. *Crt*Z and *Crt*W genes originate from a marine bacterium but were codon optimized for plant expression. It is plausible that the bacterial enzymes require cofactors less available in plants which lower their efficiency. Moreover, access to β-carotene is also certainly reduced in the plants as β-carotene is involved in the endogenous carotenoid pathway, so the heterologous enzymes need to compete for the same substrate as the endogenous hydroxylase (Fig. 1). In both hosts, all intermediates of the ketocarotenoid pathway have been detected implying that all routes to astaxanthin are metabolically active. In pZ-W *E.coli*, the reduction of adonixanthin levels suggests that the bioconversion of astaxanthin going from β-carotene through echinenone, 3-OH-echinenone, adonixanthin to astaxanthin seems enhanced by the enzyme fusion (Fig. 1). In pZ-W *benthamiana*, it is not as clear as no increase of astaxanthin content was detected. It is plausible that the decrease in canthaxanthin and phoenicoxanthin levels highlights a preference for this bioconversion route in pZ-W, with the fusion enzyme operating at a slower rate leading to no increase of astaxanthin level during the short period tested.

Potentially, an increased content of astaxanthin could be achieved by changing the stoichiometry of the enzymes. Indeed, canthaxanthin represented 50% of the intermediates in *N. benthamiana* pZ+W (Table 2), suggesting that the reaction catalyzed by CRTZ is the rate-limiting step. Therefore, changing the stoichiometry of CRTZ: CRTW to 2:1 could hypothetically increase astaxanthin levels. This could be carried out using several methods. For instance, a fusion enzyme CRTZ-CRTW-CRTZ could be built while making sure that both *Crt*Z sequences are attached by their N terminal to the *Crt*W sequence. This would require the second *Crt*Z gene to be synthesised as a reverse amino acid sequence. The hosts could be transformed in parallel with the pZ-W fusion and pZ vectors. Another technique would be to use a DNA scaffold (Fu et al., 2012, Wilner et al., 2009) or cohesion/dockerin scaffold (You et al., 2012) holding two CRTZ enzymes and one CRTW enzyme. In a different study, aiming at producing zeaxanthin in *E.coli* using *Crt*Z and *Crt*Y (lycopene cyclase) genes, the CRTZ enzyme was also considered to impose the bottleneck in the production of zeaxanthin. By changing the enzyme stoichiometry of CRTZ: CRTY to 2:1 in a non-fused manner, the production of zeaxanthin was enhanced (Li et al., 2015). The Li et al. study supports the hypothesis that modifying the stoichiometry of CRTZ: CRTW could lead to higher levels of astaxanthin. Other recent studies have shown that by localizing the (keto)carotenoid enzymes closer to the *E. coli* membrane, and thus closer to their substrates, increased levels of the (keto) carotenoid end-products could be obtained (Park et al., 2018, Wu et al., 2017). Membrane engineering is also likely to be beneficial in a plant system.

The aim of this study was to create and test a “new to nature” CRTZ-CRTW fusion enzyme in bacterial and plant hosts. We demonstrated that this fusion enzyme is capable of producing astaxanthin levels higher or similar to the free enzymes in both hosts, whilst reducing the amount of intermediates. In the present study, the levels (e.g. 0.2–0.6% dry weight) of ketocarotenoids produced transiently in *N. benthamiana* and in *E.coli* are comparable to other studies on carotenoids and isoprenoids in general, using comparable systems (Misawa and Shimada, 1998, Reed et al., 2017). In *E.coli*, levels could be greatly enhanced by optimising the *E.coli* culture conditions (strains, medium, length of time culture, chemostat; Supplementary Fig. 9). Although the ketocarotenogenic enzymes seem more efficient at converting β-carotene into astaxanthin in *E.coli*, higher plants seem a better production platform as they can accumulate higher levels of ketocarotenoids (Misawa, 2011). *Agrobacterium*-mediated transient infiltration in *N. benthamiana* is a rapid and robust plant transformation method which delivers results in weeks as opposed to the several months to years needed for the stable transformation of higher plants, requiring generation, selection and confirmation of the stable plant lines. The *N. benthamiana* transient system is successfully used for plant molecular farming. Antibodies and antigens are produced with high yields (0.5–4 g/kg of leaf fresh weight). Moreover, the scalability of the system is an attractive economic argument for such an approach, although the downstream purification burden can impact on its economic value (Yao et al., 2015). The transient tobacco system is competent at delivering quick responses to global health issues, such as the production of an Ebola vaccine during the last Ebola outbreak in 2014 (Yao et al., 2015). One caveat that should not be overlooked is that the use of *N. benthamiana* for small molecules production is still in its infancy compared to protein-based pharmaceuticals. One underlying reason is the utility of immunoaffinity purification for downstream processes. In this paper, the transient expression system via agro-infiltration has proven to be efficient to study the production of ketocarotenoids in *N. benthamiana.* A change of color in leaves was already observed after 3 days and ketocarotenoids could accumulate levels that are detectable and quantifiable in liquid chromatographic systems. This is the first time that transient production of ketocarotenoid has been reported. Producing ketocarotenoids transiently in *N. benthamiana* at an industrial level could be possible however, it would involve a lot of costly and non-sustainable downstream processes for ketocarotenoid extraction. Tomato fruits are sink organs, with GRAS properties and when engineered (overexpressing the individual *Crt*Z and *Crt*W genes), they have the ability to accumulate mg levels of ketocarotenoids (Nogueira et al., 2017). Dry powder of tomato producing ketocarotenoids, used as fish feed, have previously been shown to color trout fillets and be more efficient at retaining ketocarotenoid in the fish fillet compared to the synthetic feed (Nogueira et al., 2017). Dry tomato powder does not require any downstream processing. The production costs of this tomato material containing a kilogram of coloring ketocarotenoids (astaxanthin, phoenicoxanthin and canthaxanthin) was estimated at US$150. However, these tomatoes mainly produced canthaxanthin, phoenicoxanthin and phoenicoxanthin esters but little astaxanthin. The fusion enzyme presented in this paper could be a useful tool to channel the intermediates before they are esterified and consequently enhance accumulation of free or esterified astaxanthin in tomatoes.

A better understanding of the fusion enzyme's kinetics, reaction mechanisms and structure would be valuable assets to further improve the activity of the fusion enzyme. Protein engineering could also be employed to further engineer the catalytic potential of these fusion enzymes. Other artificial systems, such as tunnels, nanocaging, clustering or other sophisticated scaffolds, could allow a more robust confinement of the intermediates, thus preventing their escape into the bulk solution and possibly increasing the desirable end-product (Zhang and Hess, 2017).

## 5 Conclusions

This study has successfully created a range of enzyme fusions between two essential enzymatic components of the astaxanthin biosynthetic pathway, effectively creating an enzyme that is “new to nature”. Functionality has been shown in both bacterial and plant hosts, where they can produce similar levels of astaxanthin compared to the individual enzymes, whilst reducing the accumulation of intermediates. In doing so the ability of the fusion enzymes to produce astaxanthin has been improved. The quality of the plant material producing the ketocarotenoids is also enhanced due to a reduction in the stress induced by the accumulation of high levels of heterologous ketocarotenoid intermediates. The fusion of ketocarotenogenic enzymes is a promising tool to engineer astaxanthin formation in bacterial and plant hosts. This approach represents one of the first to be used for this valuable pathway and provides the impetus to explore further opportunities to construct efficient carotenoid metabolons using Synthetic Biology tools.
