# Supplementary material for: Construction of a fusion enzyme for astaxanthin formation and its characterisation in microbial and plant hosts: A new tool for engineering ketocarotenoids
Source: Metab Eng. 2019 Mar;52:243–52. doi: 10.1016/j.ymben.2018.12.006 (PMC6374281; doi:10.1016/j.ymben.2018.12.006)
Supplement: Supplementary file 1 — Supplementary material [file mmc1.pptx]

## Slide 1
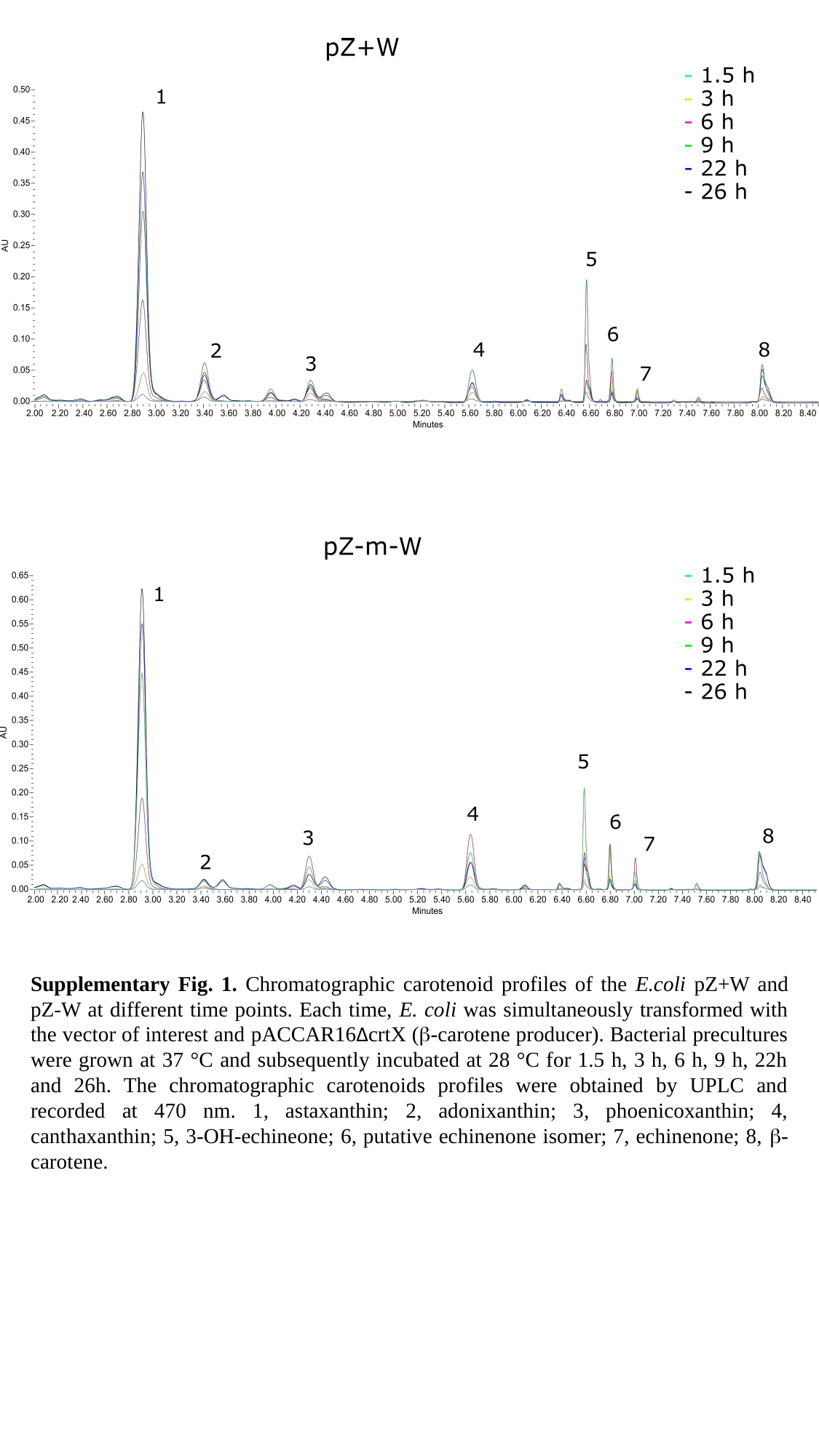

Supplementary Fig. 1. Chromatographic carotenoid profiles of the E.coli pZ+W and pZ-W at different time points. Each time, E. coli was simultaneously transformed with the vector of interest and pACCAR16ΔcrtX (β-carotene producer). Bacterial precultures were grown at 37 °C and subsequently incubated at 28 °C for 1.5 h, 3 h, 6 h, 9 h, 22h and 26h. The chromatographic carotenoids profiles were obtained by UPLC and recorded at 470 nm. 1, astaxanthin; 2, adonixanthin; 3, phoenicoxanthin; 4, canthaxanthin; 5, 3-OH-echineone; 6, putative echinenone isomer; 7, echinenone; 8, β-carotene.
