# Supplementary material for: Construction of a fusion enzyme for astaxanthin formation and its characterisation in microbial and plant hosts: A new tool for engineering ketocarotenoids
Source: Metab Eng. 2019 Mar;52:243–52. doi: 10.1016/j.ymben.2018.12.006 (PMC6374281; doi:10.1016/j.ymben.2018.12.006)
Supplement: Supplementary file 3 — Supplementary material [file mmc11.docx]

**Supplementary Table 2.** Details of the statistical tests performed with the SPSS software.

| **Display** | **analysis of** | **dependent variable** | **comparison** | | | **statistical analysis** | | **p-value** | **stars** |
| --- | --- | --- | --- | --- | --- | --- | --- | --- | --- |
| Table 2 | carotenoid | neoxanthin | pZ-s-W | compared to | p-Ø | one way ANOVA | Games-Howell | 0.532 |  |
| Table 2 | carotenoid | neoxanthin | pZ-m-W | compared to | p-Ø | one way ANOVA | Games-Howell | 0.296 |  |
| Table 2 | carotenoid | neoxanthin | pZ-lg-W | compared to | p-Ø | one way ANOVA | Games-Howell | 0.098 |  |
| Table 2 | carotenoid | neoxanthin | pZ+W | compared to | p-Ø | one way ANOVA | Games-Howell | 0.358 |  |
| Table 2 | carotenoid | violaxanthin | pZ-s-W | compared to | p-Ø | one way ANOVA | Tukey | 0.079 |  |
| Table 2 | carotenoid | violaxanthin | pZ-m-W | compared to | p-Ø | one way ANOVA | Tukey | 0.030 | * |
| Table 2 | carotenoid | violaxanthin | pZ-lg-W | compared to | p-Ø | one way ANOVA | Tukey | 0.019 | * |
| Table 2 | carotenoid | violaxanthin | pZ+W | compared to | p-Ø | one way ANOVA | Tukey | 0.005 | ** |
| Table 2 | carotenoid | zeaxanthin | pZ-s-W | compared to | p-Ø | one way ANOVA | Tukey | 0.993 |  |
| Table 2 | carotenoid | zeaxanthin | pZ-m-W | compared to | p-Ø | one way ANOVA | Tukey | 0.879 |  |
| Table 2 | carotenoid | zeaxanthin | pZ-lg-W | compared to | p-Ø | one way ANOVA | Tukey | 0.988 |  |
| Table 2 | carotenoid | zeaxanthin | pZ+W | compared to | p-Ø | one way ANOVA | Tukey | 0.971 |  |
| Table 2 | carotenoid | lutein | pZ-s-W | compared to | p-Ø | one way ANOVA | Tukey | 0.931 |  |
| Table 2 | carotenoid | lutein | pZ-m-W | compared to | p-Ø | one way ANOVA | Tukey | 0.359 |  |
| Table 2 | carotenoid | lutein | pZ-lg-W | compared to | p-Ø | one way ANOVA | Tukey | 0.062 |  |
| Table 2 | carotenoid | lutein | pZ+W | compared to | p-Ø | one way ANOVA | Tukey | 0.128 |  |
| Table 2 | carotenoid | β-carotene | pZ-s-W | compared to | p-Ø | one way ANOVA | Tukey | 0.000 | *** |
| Table 2 | carotenoid | β-carotene | pZ-m-W | compared to | p-Ø | one way ANOVA | Tukey | 0.000 | *** |
| Table 2 | carotenoid | β-carotene | pZ-lg-W | compared to | p-Ø | one way ANOVA | Tukey | 0.000 | *** |
| Table 2 | carotenoid | β-carotene | pZ+W | compared to | p-Ø | one way ANOVA | Tukey | 0.000 | *** |
| Table 2 | carotenoid | total carotenoid | pZ-s-W | compared to | p-Ø | one way ANOVA | Tukey | 0.342 |  |
| Table 2 | carotenoid | total carotenoid | pZ-m-W | compared to | p-Ø | one way ANOVA | Tukey | 0.089 |  |
| Table 2 | carotenoid | total carotenoid | pZ-lg-W | compared to | p-Ø | one way ANOVA | Tukey | 0.009 | ** |
| Table 2 | carotenoid | total carotenoid | pZ+W | compared to | p-Ø | one way ANOVA | Tukey | 0.009 | ** |
| Table 2 | ketocarotenoid | astaxanthin | pZ-s-W | compared to | pZ+W | one way ANOVA | Games-Howell | 0.464 |  |
| Table 2 | ketocarotenoid | astaxanthin | pZ-m-W | compared to | pZ+W | one way ANOVA | Games-Howell | 0.235 |  |
| Table 2 | ketocarotenoid | astaxanthin | pZ-lg-W | compared to | pZ+W | one way ANOVA | Games-Howell | 0.041 | * |
| Table 2 | ketocarotenoid | adonixanthin | pZ-s-W | compared to | pZ+W | one way ANOVA | Games-Howell | 0.382 |  |
| Table 2 | ketocarotenoid | adonixanthin | pZ-m-W | compared to | pZ+W | one way ANOVA | Games-Howell | 1.000 |  |
| Table 2 | ketocarotenoid | adonixanthin | pZ-lg-W | compared to | pZ+W | one way ANOVA | Games-Howell | 0.044 | * |
| Table 2 | ketocarotenoid | phoenicoxanthin | pZ-s-W | compared to | pZ+W | one way ANOVA | Tukey | 0.000 | *** |
| Table 2 | ketocarotenoid | phoenicoxanthin | pZ-m-W | compared to | pZ+W | one way ANOVA | Tukey | 0.001 | *** |
| Table 2 | ketocarotenoid | phoenicoxanthin | pZ-lg-W | compared to | pZ+W | one way ANOVA | Tukey | 0.000 | *** |
| Table 2 | ketocarotenoid | canthaxanthin | pZ-s-W | compared to | pZ+W | one way ANOVA | Tukey | 0.000 | *** |
| Table 2 | ketocarotenoid | canthaxanthin | pZ-m-W | compared to | pZ+W | one way ANOVA | Tukey | 0.000 | *** |
| Table 2 | ketocarotenoid | canthaxanthin | pZ-lg-W | compared to | pZ+W | one way ANOVA | Tukey | 0.000 | *** |
| Table 2 | ketocarotenoid | 3ˈ-OH-echinenone | pZ-s-W | compared to | pZ+W | one way ANOVA | Games-Howell | 0.029 | * |
| Table 2 | ketocarotenoid | 3ˈ-OH-echinenone | pZ-m-W | compared to | pZ+W | one way ANOVA | Games-Howell | 0.031 | * |
| Table 2 | ketocarotenoid | 3ˈ-OH-echinenone | pZ-lg-W | compared to | pZ+W | one way ANOVA | Games-Howell | 0.026 | * |
| Table 2 | ketocarotenoid | 3ˈ-OH-echinenone isomer | pZ-s-W | compared to | pZ+W | one way ANOVA | Games-Howell | 0.897 |  |
| Table 2 | ketocarotenoid | 3ˈ-OH-echinenone isomer | pZ-m-W | compared to | pZ+W | one way ANOVA | Games-Howell | 0.145 |  |
| Table 2 | ketocarotenoid | 3ˈ-OH-echinenone isomer | pZ-lg-W | compared to | pZ+W | one way ANOVA | Games-Howell | 0.629 |  |
| Table 2 | ketocarotenoid | echinenone | pZ-s-W | compared to | pZ+W | one way ANOVA | Games-Howell | 0.258 |  |
| Table 2 | ketocarotenoid | echinenone | pZ-m-W | compared to | pZ+W | one way ANOVA | Games-Howell | 0.818 |  |
| Table 2 | ketocarotenoid | echinenone | pZ-lg-W | compared to | pZ+W | one way ANOVA | Games-Howell | 0.446 |  |
| Table 2 | ketocarotenoid | total ketocarotenoid | pZ-s-W | compared to | pZ+W | one way ANOVA | Games-Howell | 0.000 | *** |
| Table 2 | ketocarotenoid | total ketocarotenoid | pZ-m-W | compared to | pZ+W | one way ANOVA | Games-Howell | 0.000 | *** |
| Table 2 | ketocarotenoid | total ketocarotenoid | pZ-lg-W | compared to | pZ+W | one way ANOVA | Games-Howell | 0.000 | *** |
| Table 2 | carotenoid | total | pZ-s-W | compared to | p-Ø | one way ANOVA | Tukey | 0.744 |  |
| Table 2 | carotenoid | total | pZ-m-W | compared to | p-Ø | one way ANOVA | Tukey | 0.346 |  |
| Table 2 | carotenoid | total | pZ-lg-W | compared to | p-Ø | one way ANOVA | Tukey | 0.032 | * |
| Table 2 | carotenoid | total | pZ+W | compared to | p-Ø | one way ANOVA | Tukey | 0.161 |  |
| Table 1 | ketocarotenoid_ 1.5 h | astaxanthin | pZ+W | compared to | pZ-m-W | t-test | | 0.045 | * |
| Table 1 | ketocarotenoid_ 1.5 h | adonixanthin | pZ+W | compared to | pZ-m-W | t-test | | 0.159 |  |
| Table 1 | ketocarotenoid_ 1.5 h | phoenicoxanthin | pZ+W | compared to | pZ-m-W | t-test | | 0.086 |  |
| Table 1 | ketocarotenoid_ 1.5 h | canthaxanthin | pZ+W | compared to | pZ-m-W | t-test | | 0.169 |  |
| Table 1 | ketocarotenoid_ 1.5 h | 3-OH-echinenone | pZ+W | compared to | pZ-m-W | t-test | | 0.005 | ** |
| Table 1 | ketocarotenoid_ 1.5 h | echinenone & isomer | pZ+W | compared to | pZ-m-W | t-test | | 0.032 |  |
| Table 1 | carotenoid_ 1.5 h | β-carotene | pZ+W | compared to | pZ-m-W | t-test | | 0.032 |  |
| Table 1 | ketocarotenoid_ 1.5 h | Total ketocarotenoid | pZ+W | compared to | pZ-m-W | t-test | | 0.045 |  |
| Table 1 | carotenoid_ 1.5 h | Total | pZ+W | compared to | pZ-m-W | t-test | | 0.039 |  |
| Table 1 | ketocarotenoid_ 3 h | astaxanthin | pZ+W | compared to | pZ-m-W | t-test | | 0.292 |  |
| Table 1 | ketocarotenoid_ 3 h | adonixanthin | pZ+W | compared to | pZ-m-W | t-test | | 0.326 |  |
| Table 1 | ketocarotenoid_ 3 h | phoenicoxanthin | pZ+W | compared to | pZ-m-W | t-test | | 0.001 | *** |
| Table 1 | ketocarotenoid_ 3 h | canthaxanthin | pZ+W | compared to | pZ-m-W | t-test | | 0.053 |  |
| Table 1 | ketocarotenoid_ 3 h | 3-OH-echinenone | pZ+W | compared to | pZ-m-W | t-test | | 0.999 |  |
| Table 1 | ketocarotenoid_ 3 h | echinenone & isomer | pZ+W | compared to | pZ-m-W | t-test | | 0.446 |  |
| Table 1 | carotenoid_ 3 h | β-carotene | pZ+W | compared to | pZ-m-W | t-test | | 0.071 |  |
| Table 1 | ketocarotenoid_ 3 h | Total ketocarotenoid | pZ+W | compared to | pZ-m-W | t-test | | 0.261 |  |
| Table 1 | carotenoid_ 3 h | Total | pZ+W | compared to | pZ-m-W | t-test | | 0.175 |  |
| Table 1 | ketocarotenoid_ 6 h | astaxanthin | pZ+W | compared to | pZ-m-W | t-test | | 0.483 |  |
| Table 1 | ketocarotenoid_ 6 h | adonixanthin | pZ+W | compared to | pZ-m-W | t-test | | 0.000 | *** |
| Table 1 | ketocarotenoid_ 6 h | phoenicoxanthin | pZ+W | compared to | pZ-m-W | t-test | | 0.262 |  |
| Table 1 | ketocarotenoid_ 6 h | canthaxanthin | pZ+W | compared to | pZ-m-W | t-test | | 0.125 |  |
| Table 1 | ketocarotenoid_ 6 h | 3-OH-echinenone | pZ+W | compared to | pZ-m-W | t-test | | 0.012 | * |
| Table 1 | ketocarotenoid_ 6 h | echinenone & isomer | pZ+W | compared to | pZ-m-W | t-test | | 0.882 |  |
| Table 1 | carotenoid_ 6 h | β-carotene | pZ+W | compared to | pZ-m-W | t-test | | 0.953 |  |
| Table 1 | ketocarotenoid_ 6 h | Total ketocarotenoid | pZ+W | compared to | pZ-m-W | t-test | | 0.509 |  |
| Table 1 | carotenoid_ 6 h | Total | pZ+W | compared to | pZ-m-W | t-test | | 0.546 |  |
| Table 1 | ketocarotenoid_ 9 h | astaxanthin | pZ+W | compared to | pZ-m-W | t-test | | 0.013 | * |
| Table 1 | ketocarotenoid_ 9 h | adonixanthin | pZ+W | compared to | pZ-m-W | t-test | | 0.002 | ** |
| Table 1 | ketocarotenoid_ 9 h | phoenicoxanthin | pZ+W | compared to | pZ-m-W | t-test | | 0.543 |  |
| Table 1 | ketocarotenoid_ 9 h | canthaxanthin | pZ+W | compared to | pZ-m-W | t-test | | 0.809 |  |
| Table 1 | ketocarotenoid_ 9 h | 3-OH-echinenone | pZ+W | compared to | pZ-m-W | t-test | | 0.148 |  |
| Table 1 | ketocarotenoid_ 9 h | echinenone & isomer | pZ+W | compared to | pZ-m-W | t-test | | 0.557 |  |
| Table 1 | carotenoid_ 9 h | β-carotene | pZ+W | compared to | pZ-m-W | t-test | | 0.085 |  |
| Table 1 | ketocarotenoid_ 9 h | Total ketocarotenoid | pZ+W | compared to | pZ-m-W | t-test | | 0.581 |  |
| Table 1 | carotenoid_ 9 h | Total | pZ+W | compared to | pZ-m-W | t-test | | 0.412 |  |
| Table 1 | ketocarotenoid_ 22 h | astaxanthin | pZ+W | compared to | pZ-m-W | t-test | | 0.000 | *** |
| Table 1 | ketocarotenoid_ 22 h | adonixanthin | pZ+W | compared to | pZ-m-W | t-test | | 0.000 | *** |
| Table 1 | ketocarotenoid_ 22 h | phoenicoxanthin | pZ+W | compared to | pZ-m-W | t-test | | 0.465 |  |
| Table 1 | ketocarotenoid_ 22 h | canthaxanthin | pZ+W | compared to | pZ-m-W | t-test | | 0.825 |  |
| Table 1 | ketocarotenoid_ 22 h | 3-OH-echinenone | pZ+W | compared to | pZ-m-W | t-test | | 0.306 |  |
| Table 1 | ketocarotenoid_ 22 h | echinenone & isomer | pZ+W | compared to | pZ-m-W | t-test | | 0.229 |  |
| Table 1 | carotenoid_ 22 h | β-carotene | pZ+W | compared to | pZ-m-W | t-test | | 0.003 | ** |
| Table 1 | ketocarotenoid_ 22 h | Total ketocarotenoid | pZ+W | compared to | pZ-m-W | t-test | | 0.008 | ** |
| Table 1 | carotenoid_ 22 h | Total | pZ+W | compared to | pZ-m-W | t-test | | 0.001 | *** |
| Table 1 | ketocarotenoid_ 26 h | astaxanthin | pZ+W | compared to | pZ-m-W | t-test | | 0.025 | * |
| Table 1 | ketocarotenoid_ 26 h | adonixanthin | pZ+W | compared to | pZ-m-W | t-test | | 0.002 | ** |
| Table 1 | ketocarotenoid_ 26 h | phoenicoxanthin | pZ+W | compared to | pZ-m-W | t-test | | 0.246 |  |
| Table 1 | ketocarotenoid_ 26 h | canthaxanthin | pZ+W | compared to | pZ-m-W | t-test | | 0.554 |  |
| Table 1 | ketocarotenoid_ 26 h | 3-OH-echinenone | pZ+W | compared to | pZ-m-W | t-test | | 0.971 |  |
| Table 1 | ketocarotenoid_ 26 h | echinenone & isomer | pZ+W | compared to | pZ-m-W | t-test | | 0.902 |  |
| Table 1 | carotenoid_ 26 h | β-carotene | pZ+W | compared to | pZ-m-W | t-test | | 0.006 | ** |
| Table 1 | ketocarotenoid_ 26 h | Total ketocarotenoid | pZ+W | compared to | pZ-m-W | t-test | | 0.287 |  |
| Table 1 | carotenoid_ 26 h | Total | pZ+W | compared to | pZ-m-W | t-test | | 0.102 |  |
| Supplementary Table 4 | pZ+W | astaxanthin | at 1.5 h | compared to | at 3 h | repeated measures ANOVA | | 1 |  |
| Supplementary Table 4 | pZ+W | astaxanthin | at 3 h | compared to | at 6 h | repeated measures ANOVA | | 0.405 |  |
| Supplementary Table 4 | pZ+W | astaxanthin | at 6 h | compared to | at 9 h | repeated measures ANOVA | | 0.058 |  |
| Supplementary Table 4 | pZ+W | astaxanthin | at 9 h | compared to | at 22 h | repeated measures ANOVA | | 0.083 |  |
| Supplementary Table 4 | pZ+W | astaxanthin | at 22 h | compared to | at 26 h | repeated measures ANOVA | | 1 |  |
| Supplementary Table 4 | pZ+W | adonixanthin | at 1.5 h | compared to | at 3 h | repeated measures ANOVA | | 0.469 |  |
| Supplementary Table 4 | pZ+W | adonixanthin | at 3 h | compared to | at 6 h | repeated measures ANOVA | | 0.687 |  |
| Supplementary Table 4 | pZ+W | adonixanthin | at 6 h | compared to | at 9 h | repeated measures ANOVA | | 0.276 |  |
| Supplementary Table 4 | pZ+W | adonixanthin | at 9 h | compared to | at 22 h | repeated measures ANOVA | | 0.382 |  |
| Supplementary Table 4 | pZ+W | adonixanthin | at 22 h | compared to | at 26 h | repeated measures ANOVA | | 1 |  |
| Supplementary Table 4 | pZ+W | phoenicoxanthin | at 1.5 h | compared to | at 3 h | repeated measures ANOVA | | 0.101 |  |
| Supplementary Table 4 | pZ+W | phoenicoxanthin | at 3 h | compared to | at 6 h | repeated measures ANOVA | | 1 |  |
| Supplementary Table 4 | pZ+W | phoenicoxanthin | at 6 h | compared to | at 9 h | repeated measures ANOVA | | 0.120 |  |
| Supplementary Table 4 | pZ+W | phoenicoxanthin | at 9 h | compared to | at 22 h | repeated measures ANOVA | | 1 |  |
| Supplementary Table 4 | pZ+W | phoenicoxanthin | at 22 h | compared to | at 26 h | repeated measures ANOVA | | 1 |  |
| Supplementary Table 4 | pZ+W | canthaxanthin | at 1.5 h | compared to | at 3 h | repeated measures ANOVA | | 0.299 |  |
| Supplementary Table 4 | pZ+W | canthaxanthin | at 3 h | compared to | at 6 h | repeated measures ANOVA | | 1 |  |
| Supplementary Table 4 | pZ+W | canthaxanthin | at 6 h | compared to | at 9 h | repeated measures ANOVA | | 0.807 |  |
| Supplementary Table 4 | pZ+W | canthaxanthin | at 9 h | compared to | at 22 h | repeated measures ANOVA | | 1 |  |
| Supplementary Table 4 | pZ+W | canthaxanthin | at 22 h | compared to | at 26 h | repeated measures ANOVA | | 1 |  |
| Supplementary Table 4 | pZ+W | 3-OH-echinenone | at 1.5 h | compared to | at 3 h | repeated measures ANOVA | | 0.072 |  |
| Supplementary Table 4 | pZ+W | 3-OH-echinenone | at 3 h | compared to | at 6 h | repeated measures ANOVA | | 1 |  |
| Supplementary Table 4 | pZ+W | 3-OH-echinenone | at 6 h | compared to | at 9 h | repeated measures ANOVA | | 0.038 | * |
| Supplementary Table 4 | pZ+W | 3-OH-echinenone | at 9 h | compared to | at 22 h | repeated measures ANOVA | | 0.159 |  |
| Supplementary Table 4 | pZ+W | 3-OH-echinenone | at 22 h | compared to | at 26 h | repeated measures ANOVA | | 1 |  |
| Supplementary Table 4 | pZ+W | echinenone & isomer | at 1.5 h | compared to | at 3 h | repeated measures ANOVA | | 0.059 |  |
| Supplementary Table 4 | pZ+W | echinenone & isomer | at 3 h | compared to | at 6 h | repeated measures ANOVA | | 1 |  |
| Supplementary Table 4 | pZ+W | echinenone & isomer | at 6 h | compared to | at 9 h | repeated measures ANOVA | | 0.203 |  |
| Supplementary Table 4 | pZ+W | echinenone & isomer | at 9 h | compared to | at 22 h | repeated measures ANOVA | | 0.993 |  |
| Supplementary Table 4 | pZ+W | echinenone & isomer | at 22 h | compared to | at 26 h | repeated measures ANOVA | | 1 |  |
| Supplementary Table 4 | pZ+W | β-carotene | at 1.5 h | compared to | at 3 h | repeated measures ANOVA | | 0.060 |  |
| Supplementary Table 4 | pZ+W | β-carotene | at 3 h | compared to | at 6 h | repeated measures ANOVA | | 1 |  |
| Supplementary Table 4 | pZ+W | β-carotene | at 6 h | compared to | at 9 h | repeated measures ANOVA | | 0.970 |  |
| Supplementary Table 4 | pZ+W | β-carotene | at 9 h | compared to | at 22 h | repeated measures ANOVA | | 1 |  |
| Supplementary Table 4 | pZ+W | β-carotene | at 22 h | compared to | at 26 h | repeated measures ANOVA | | 1 |  |
| Supplementary Table 4 | pZ+W | Total ketocarotenoid | at 1.5 h | compared to | at 3 h | repeated measures ANOVA | | 0.217 |  |
| Supplementary Table 4 | pZ+W | Total ketocarotenoid | at 3 h | compared to | at 6 h | repeated measures ANOVA | | 1 |  |
| Supplementary Table 4 | pZ+W | Total ketocarotenoid | at 6 h | compared to | at 9 h | repeated measures ANOVA | | 0.085 |  |
| Supplementary Table 4 | pZ+W | Total ketocarotenoid | at 9 h | compared to | at 22 h | repeated measures ANOVA | | 1 |  |
| Supplementary Table 4 | pZ+W | Total ketocarotenoid | at 22 h | compared to | at 26 h | repeated measures ANOVA | | 1 |  |
| Supplementary Table 4 | pZ+W | Total | at 1.5 h | compared to | at 3 h | repeated measures ANOVA | | 0.146 |  |
| Supplementary Table 4 | pZ+W | Total | at 3 h | compared to | at 6 h | repeated measures ANOVA | | 1 |  |
| Supplementary Table 4 | pZ+W | Total | at 6 h | compared to | at 9 h | repeated measures ANOVA | | 0.055 |  |
| Supplementary Table 4 | pZ+W | Total | at 9 h | compared to | at 22 h | repeated measures ANOVA | | 1 |  |
| Supplementary Table 4 | pZ+W | Total | at 22 h | compared to | at 26 h | repeated measures ANOVA | | 1 |  |
| Supplementary Table 4 | pZ-m-W | astaxanthin | at 1.5 h | compared to | at 3 h | repeated measures ANOVA | | 1 |  |
| Supplementary Table 4 | pZ-m-W | astaxanthin | at 3 h | compared to | at 6 h | repeated measures ANOVA | | 0.344 |  |
| Supplementary Table 4 | pZ-m-W | astaxanthin | at 6 h | compared to | at 9 h | repeated measures ANOVA | | 0.078 |  |
| Supplementary Table 4 | pZ-m-W | astaxanthin | at 9 h | compared to | at 22 h | repeated measures ANOVA | | 0.108 |  |
| Supplementary Table 4 | pZ-m-W | astaxanthin | at 22 h | compared to | at 26 h | repeated measures ANOVA | | 1 |  |
| Supplementary Table 4 | pZ-m-W | adonixanthin | at 1.5 h | compared to | at 3 h | repeated measures ANOVA | | 0.094 |  |
| Supplementary Table 4 | pZ-m-W | adonixanthin | at 3 h | compared to | at 6 h | repeated measures ANOVA | | 1 |  |
| Supplementary Table 4 | pZ-m-W | adonixanthin | at 6 h | compared to | at 9 h | repeated measures ANOVA | | 0.924 |  |
| Supplementary Table 4 | pZ-m-W | adonixanthin | at 9 h | compared to | at 22 h | repeated measures ANOVA | | 1 |  |
| Supplementary Table 4 | pZ-m-W | adonixanthin | at 22 h | compared to | at 26 h | repeated measures ANOVA | | 0.130 |  |
| Supplementary Table 4 | pZ-m-W | phoenicoxanthin | at 1.5 h | compared to | at 3 h | repeated measures ANOVA | | 0.125 |  |
| Supplementary Table 4 | pZ-m-W | phoenicoxanthin | at 3 h | compared to | at 6 h | repeated measures ANOVA | | 1 |  |
| Supplementary Table 4 | pZ-m-W | phoenicoxanthin | at 6 h | compared to | at 9 h | repeated measures ANOVA | | 1 |  |
| Supplementary Table 4 | pZ-m-W | phoenicoxanthin | at 9 h | compared to | at 22 h | repeated measures ANOVA | | 1 |  |
| Supplementary Table 4 | pZ-m-W | phoenicoxanthin | at 22 h | compared to | at 26 h | repeated measures ANOVA | | 1 |  |
| Supplementary Table 4 | pZ-m-W | canthaxanthin | at 1.5 h | compared to | at 3 h | repeated measures ANOVA | | 0.383 |  |
| Supplementary Table 4 | pZ-m-W | canthaxanthin | at 3 h | compared to | at 6 h | repeated measures ANOVA | | 1 |  |
| Supplementary Table 4 | pZ-m-W | canthaxanthin | at 6 h | compared to | at 9 h | repeated measures ANOVA | | 1 |  |
| Supplementary Table 4 | pZ-m-W | canthaxanthin | at 9 h | compared to | at 22 h | repeated measures ANOVA | | 1 |  |
| Supplementary Table 4 | pZ-m-W | canthaxanthin | at 22 h | compared to | at 26 h | repeated measures ANOVA | | 1 |  |
| Supplementary Table 4 | pZ-m-W | 3ˈ-OH-echinenone | at 1.5 h | compared to | at 3 h | repeated measures ANOVA | | 0.069 |  |
| Supplementary Table 4 | pZ-m-W | 3ˈ-OH-echinenone | at 3 h | compared to | at 6 h | repeated measures ANOVA | | 1 |  |
| Supplementary Table 4 | pZ-m-W | 3ˈ-OH-echinenone | at 6 h | compared to | at 9 h | repeated measures ANOVA | | 0.069 |  |
| Supplementary Table 4 | pZ-m-W | 3ˈ-OH-echinenone | at 9 h | compared to | at 22 h | repeated measures ANOVA | | 0.499 |  |
| Supplementary Table 4 | pZ-m-W | 3ˈ-OH-echinenone | at 22 h | compared to | at 26 h | repeated measures ANOVA | | 1 |  |
| Supplementary Table 4 | pZ-m-W | echinenone & isomer | at 1.5 h | compared to | at 3 h | repeated measures ANOVA | | 0.084 |  |
| Supplementary Table 4 | pZ-m-W | echinenone & isomer | at 3 h | compared to | at 6 h | repeated measures ANOVA | | 1 |  |
| Supplementary Table 4 | pZ-m-W | echinenone & isomer | at 6 h | compared to | at 9 h | repeated measures ANOVA | | 1 |  |
| Supplementary Table 4 | pZ-m-W | echinenone & isomer | at 9 h | compared to | at 22 h | repeated measures ANOVA | | 0.724 |  |
| Supplementary Table 4 | pZ-m-W | echinenone & isomer | at 22 h | compared to | at 26 h | repeated measures ANOVA | | 1 |  |
| Supplementary Table 4 | pZ-m-W | β-carotene | at 1.5 h | compared to | at 3 h | repeated measures ANOVA | | 0.095 |  |
| Supplementary Table 4 | pZ-m-W | β-carotene | at 3 h | compared to | at 6 h | repeated measures ANOVA | | 0.729 |  |
| Supplementary Table 4 | pZ-m-W | β-carotene | at 6 h | compared to | at 9 h | repeated measures ANOVA | | 1 |  |
| Supplementary Table 4 | pZ-m-W | β-carotene | at 9 h | compared to | at 22 h | repeated measures ANOVA | | 1 |  |
| Supplementary Table 4 | pZ-m-W | β-carotene | at 22 h | compared to | at 26 h | repeated measures ANOVA | | 1 |  |
| Supplementary Table 4 | pZ-m-W | Total ketocarotenoid | at 1.5 h | compared to | at 3 h | repeated measures ANOVA | | 0.177 |  |
| Supplementary Table 4 | pZ-m-W | Total ketocarotenoid | at 3 h | compared to | at 6 h | repeated measures ANOVA | | 0.221 |  |
| Supplementary Table 4 | pZ-m-W | Total ketocarotenoid | at 6 h | compared to | at 9 h | repeated measures ANOVA | | 0.199 |  |
| Supplementary Table 4 | pZ-m-W | Total ketocarotenoid | at 9 h | compared to | at 22 h | repeated measures ANOVA | | 1 |  |
| Supplementary Table 4 | pZ-m-W | Total ketocarotenoid | at 22 h | compared to | at 26 h | repeated measures ANOVA | | 1 |  |
| Supplementary Table 4 | pZ-m-W | Total | at 1.5 h | compared to | at 3 h | repeated measures ANOVA | | 0.143 |  |
| Supplementary Table 4 | pZ-m-W | Total | at 3 h | compared to | at 6 h | repeated measures ANOVA | | 0.533 |  |
| Supplementary Table 4 | pZ-m-W | Total | at 6 h | compared to | at 9 h | repeated measures ANOVA | | 0.268 |  |
| Supplementary Table 4 | pZ-m-W | Total | at 9 h | compared to | at 22 h | repeated measures ANOVA | | 1 |  |
| Supplementary Table 4 | pZ-m-W | Total | at 22 h | compared to | at 26 h | repeated measures ANOVA | | 1 |  |
