# Supplementary material for: Construction of a fusion enzyme for astaxanthin formation and its characterisation in microbial and plant hosts: A new tool for engineering ketocarotenoids
Source: Metab Eng. 2019 Mar;52:243–52. doi: 10.1016/j.ymben.2018.12.006 (PMC6374281; doi:10.1016/j.ymben.2018.12.006)
Supplement: Supplementary file 4 — Supplementary material [file mmc12.docx]

**Supplementary Table 3.** Carotenoid content in *E.coli* expressing the different fusion and control constructs. *E. coli* was simultaneously transformed with the vector of interest and the pACCAR16ΔcrtX (β-carotene producer), except for p-Ø, which corresponds only to pACCAR16ΔcrtX. Carotenoid levels are represented as mg/L of culture, n=2. The mean data are shown as ± SD. Nd and nq signify that a compound has not been detected or has been detected but is under the limit of quantification, respectively.
