# Supplementary material for: Construction of a fusion enzyme for astaxanthin formation and its characterisation in microbial and plant hosts: A new tool for engineering ketocarotenoids
Source: Metab Eng. 2019 Mar;52:243–52. doi: 10.1016/j.ymben.2018.12.006 (PMC6374281; doi:10.1016/j.ymben.2018.12.006)
Supplement: Supplementary file 5 — Supplementary material [file mmc13.docx]

**Supplementary Table 4.** Carotenoid titer in pZ+W (individual) and pZ-W (fusion) *E.coli* over six time points. *E. coli* was simultaneously transformed with the vector of interest and the pACCAR16ΔcrtX (β-carotene producer). Bacterial precultures were grown at 37 °C and subsequently incubated at 28 °C for 1.5 h, 3 h, 6 h, 9 h, 22 h and 26 h. Carotenoid levels are represented as mg/L, n=3. The mean data are shown as ± SD.
