# Supplementary material for: Construction of a fusion enzyme for astaxanthin formation and its characterisation in microbial and plant hosts: A new tool for engineering ketocarotenoids
Source: Metab Eng. 2019 Mar;52:243–52. doi: 10.1016/j.ymben.2018.12.006 (PMC6374281; doi:10.1016/j.ymben.2018.12.006)
Supplement: Supplementary file 6 — Supplementary material [file mmc14.docx]

|  |  | p-Ø | | | | pZ+W | | | pZ-s-W | | | pZ-m-W | | | pZ-lg-W | | |
| --- | --- | --- | --- | --- | --- | --- | --- | --- | --- | --- | --- | --- | --- | --- | --- | --- | --- |
| Caro. | Neoxanthin | | 446.0 | ± | 68.8 | 388.0 | ± | 78.9 | 450.6 | ± | 43.1 | 496.3 | ± | 26.3 | 448.5 | ± | 43.0 |
|  | Violaxanthin | | 502.1 | ± | 69.2 | **352.0** | **±** | **56.9*** | 478.7 | ± | 62.8 | 509.8 | ± | 32.2 | 480.2 | ± | 60.1 |
|  | Zeaxanthin | | 236.1 | ± | 16.3 | 260.4 | ± | 23.3 | 256.0 | ± | 12.2 | 258.8 | ± | 9.7 | 241.7 | ± | 5.3 |
|  | Lutein | | 965.9 | ± | 194.2 | 844.6 | ± | 208.5 | 1021.5 | ± | 122.9 | 1133.9 | ± | 75.3 | 1015.3 | ± | 115.5 |
|  | β-carotene | | 292.3 | ± | 73.2 | **87.5** | **±** | **8.8***** | 207.9 | ± | 43.3 | 217.8 | ± | 23.2 | 222.3 | ± | 30.0 |
|  | Total carotenoid | | 2442.4 | ± | 392.3 | 1932.4 | ± | 368.9 | 2414.8 | ± | 238.1 | 2616.5 | ± | 148.3 | 2408.1 | ± | 225.9 |
| Keto. | Astaxanthin | | nd | | | 22.2 | ± | 3.1 | **5.8** | **±** | **3.1*** | **3.3** | **±** | **1.5**** | **2.5** | **±** | **1.3**** |
|  | Adonixanthin | | nd | | | 5.5 | ± | 6.8 | nq | | | nq | | | nq | | |
|  | Phoenicoxanthin | | nd | | | 74.3 | ± | 26.3 | **10.0** | **±** | **1.8***** | **8.6** | **±** | **0.4***** | **7.9** | **±** | **1.1***** |
|  | Canthaxanthin | | nd | | | 254.1 | ± | 70.7 | **7.2** | **±** | **5.4***** | **9.6** | **±** | **2.5***** | **4.0** | **±** | **3.6***** |
|  | 3´-OH-Echi. isomer | | nd | | | 26.1 | ± | 9.8 | 36.4 | ± | 8.7 | 38.5 | ± | 2.3 | 29.5 | ± | 4.9 |
|  | Echinenone | | nd | | | 30.3 | ± | 9.3 | 31.8 | ± | 2.0 | 30.0 | ± | 4.0 | 27.7 | ± | 3.7 |
|  | Total ketocarotenoid | | nd | | | 412.5 | ± | 129.8 | **91.2** | **±** | **22.1***** | **90.1** | **±** | **8.1***** | **71.6** | **±** | **14.8***** |
|  | Total | 2442.4 | | ± | 392.3 | 2426.9 | ± | 495.7 | 2528.5 | ± | 245.4 | 2725.2 | ± | 155.0 | 2498.4 | ± | 238.3 |

**Supplementary Table 5.** Carotenoid content in agro-infiltrated *N. benthamiana* leaves collected at 5 dpi (experiment 1). Carotenoid levels are represented as µg/g dry weight. Three leaves were pooled per plant and 3 plants were used for each agro-infiltration. Three determinations were then made per plant. The mean data are shown as ± SD. Nd signifies that a compound has not been detected and nq that a compound has been detected but is under the limit of quantification. Values in bold indicate where significant differences have been found when comparing pZ+W and pZ-W to p-Ø for the carotenoids and pZ-W to pZ+W for the ketocarotenoids. P<0.05, P<0.01 and P<0.001 are designated by *****,****,** and *******, respectively. Caro., carotenoids; Keto., ketocarotenoids. Computed p-values are tabulated in Supplementary Table 2.
