# Supplementary material for: Construction of a fusion enzyme for astaxanthin formation and its characterisation in microbial and plant hosts: A new tool for engineering ketocarotenoids
Source: Metab Eng. 2019 Mar;52:243–52. doi: 10.1016/j.ymben.2018.12.006 (PMC6374281; doi:10.1016/j.ymben.2018.12.006)
Supplement: Supplementary file 7 — Supplementary material [file mmc2.pptx]

## Slide 1
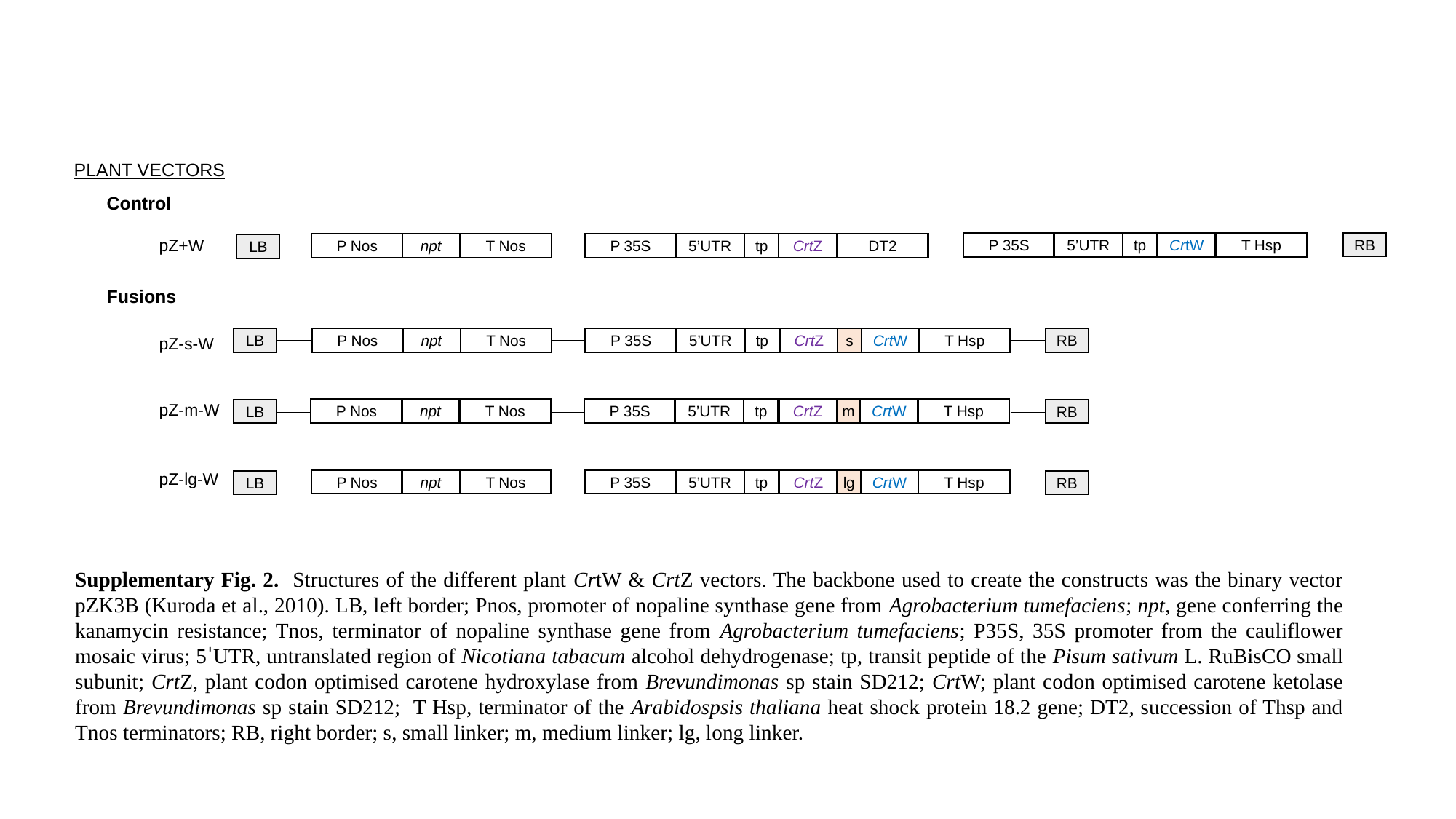

PLANT VECTORS
Control
pZ+W
RB
P 35S
5’UTR
tp
CrtW
T Hsp
P Nos
npt
T Nos
P 35S
5’UTR
tp
CrtZ
DT2
LB
Fusions
pZ-s-W
LB
P Nos
npt
T Nos
P 35S
5’UTR
tp
CrtZ
s
CrtW
T Hsp
RB
pZ-m-W
P Nos
npt
T Nos
P 35S
5’UTR
tp
CrtZ
m
CrtW
T Hsp
LB
RB
pZ-lg-W
P Nos
npt
T Nos
P 35S
5’UTR
tp
CrtZ
lg
CrtW
T Hsp
LB
RB
Supplementary Fig. 2. Structures of the different plant CrtW & CrtZ vectors. The backbone used to create the constructs was the binary vector pZK3B (Kuroda et al., 2010). LB, left border; Pnos, promoter of nopaline synthase gene from Agrobacterium tumefaciens; npt, gene conferring the kanamycin resistance; Tnos, terminator of nopaline synthase gene from Agrobacterium tumefaciens; P35S, 35S promoter from the cauliflower mosaic virus; 5ˈUTR, untranslated region of Nicotiana tabacum alcohol dehydrogenase; tp, transit peptide of the Pisum sativum L. RuBisCO small subunit; CrtZ, plant codon optimised carotene hydroxylase from Brevundimonas sp stain SD212; CrtW; plant codon optimised carotene ketolase from Brevundimonas sp stain SD212; T Hsp, terminator of the Arabidospsis thaliana heat shock protein 18.2 gene; DT2, succession of Thsp and Tnos terminators; RB, right border; s, small linker; m, medium linker; lg, long linker.
