# Supplementary material for: Construction of a fusion enzyme for astaxanthin formation and its characterisation in microbial and plant hosts: A new tool for engineering ketocarotenoids
Source: Metab Eng. 2019 Mar;52:243–52. doi: 10.1016/j.ymben.2018.12.006 (PMC6374281; doi:10.1016/j.ymben.2018.12.006)
Supplement: Supplementary file 8 — Supplementary material [file mmc3.pptx]

## Slide 1
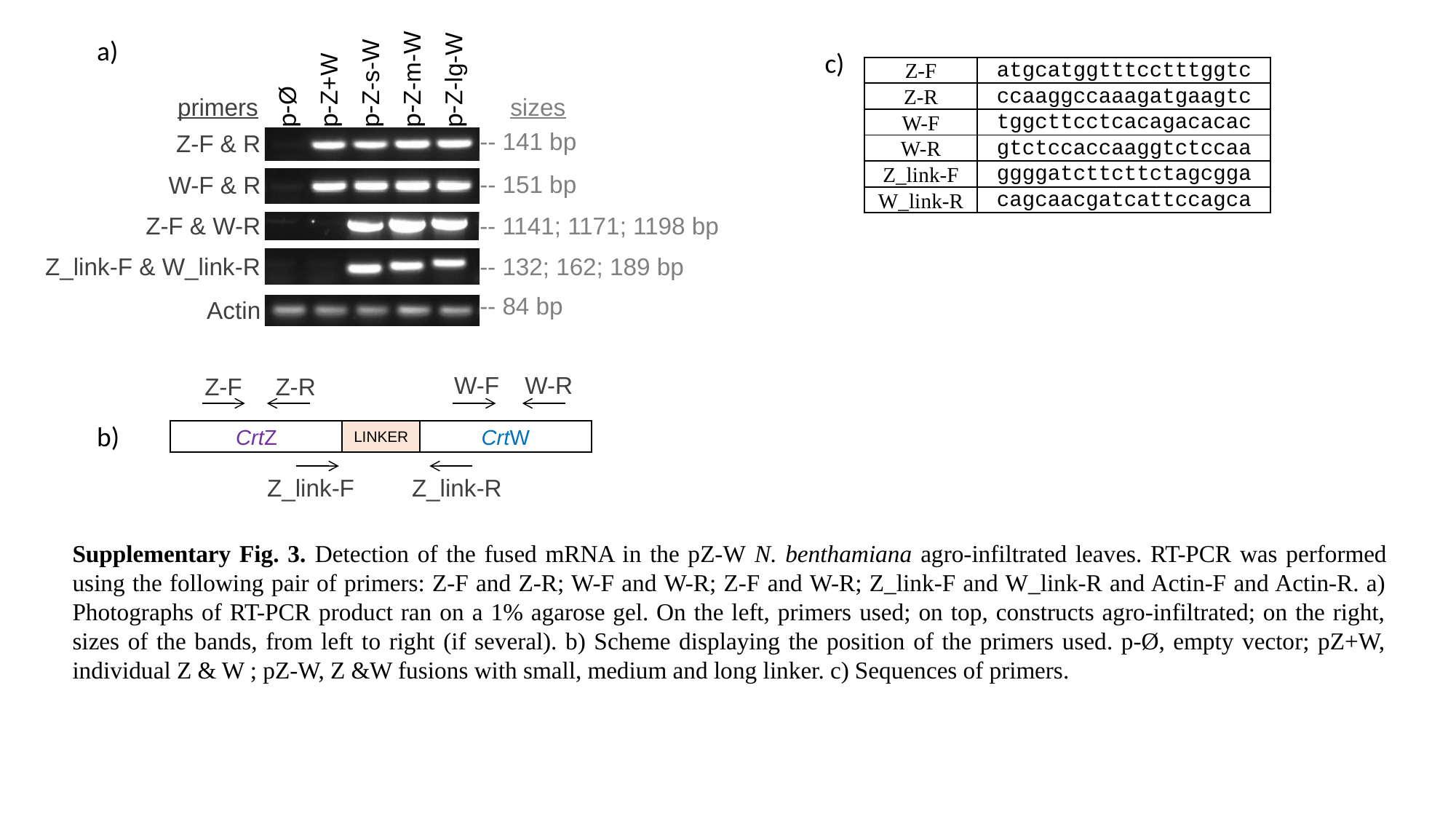

a)
c)
p-Z-m-W
p-Z-lg-W
p-Z+W
p-Z-s-W
| Z-F | atgcatggtttcctttggtc |
| --- | --- |
| Z-R | ccaaggccaaagatgaagtc |
| W-F | tggcttcctcacagacacac |
| W-R | gtctccaccaaggtctccaa |
| Z\_link-F | ggggatcttcttctagcgga |
| W\_link-R | cagcaacgatcattccagca |
p-Ø
primers
sizes
-- 141 bp
Z-F & R
-- 151 bp
W-F & R
Z-F & W-R
-- 1141; 1171; 1198 bp
-- 132; 162; 189 bp
Z_link-F & W_link-R
-- 84 bp
Actin
W-F
W-R
Z-F
Z-R
CrtZ
LINKER
CrtW
Z_link-F
Z_link-R
b)
Supplementary Fig. 3. Detection of the fused mRNA in the pZ-W N. benthamiana agro-infiltrated leaves. RT-PCR was performed using the following pair of primers: Z-F and Z-R; W-F and W-R; Z-F and W-R; Z_link-F and W_link-R and Actin-F and Actin-R. a) Photographs of RT-PCR product ran on a 1% agarose gel. On the left, primers used; on top, constructs agro-infiltrated; on the right, sizes of the bands, from left to right (if several). b) Scheme displaying the position of the primers used. p-Ø, empty vector; pZ+W, individual Z & W ; pZ-W, Z &W fusions with small, medium and long linker. c) Sequences of primers.
