# Supplementary material for: Construction of a fusion enzyme for astaxanthin formation and its characterisation in microbial and plant hosts: A new tool for engineering ketocarotenoids
Source: Metab Eng. 2019 Mar;52:243–52. doi: 10.1016/j.ymben.2018.12.006 (PMC6374281; doi:10.1016/j.ymben.2018.12.006)
Supplement: Supplementary file 9 — Supplementary material [file mmc4.pptx]

## Slide 1
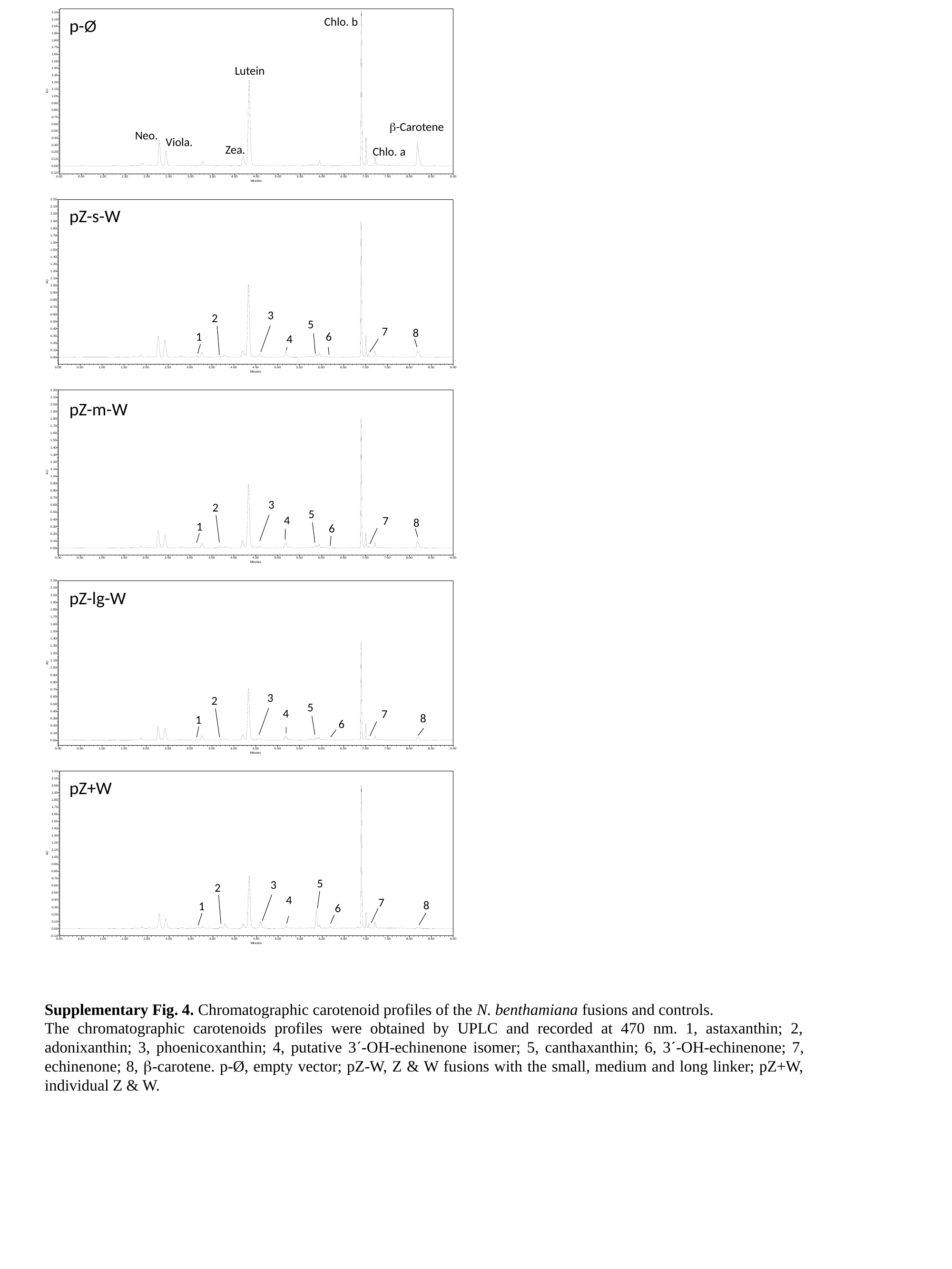

p-Ø
Chlo. b
Lutein
β-Carotene
Neo.
Viola.
Zea.
Chlo. a
pZ-s-W
3
2
5
7
8
6
1
4
pZ-m-W
3
2
5
4
7
8
1
6
pZ-lg-W
3
2
5
4
7
8
1
6
pZ+W
5
3
2
4
7
8
1
6
Supplementary Fig. 4. Chromatographic carotenoid profiles of the N. benthamiana fusions and controls.
The chromatographic carotenoids profiles were obtained by UPLC and recorded at 470 nm. 1, astaxanthin; 2, adonixanthin; 3, phoenicoxanthin; 4, putative 3´-OH-echinenone isomer; 5, canthaxanthin; 6, 3´-OH-echinenone; 7, echinenone; 8, β-carotene. p-Ø, empty vector; pZ-W, Z & W fusions with the small, medium and long linker; pZ+W, individual Z & W.
