# Supplementary material for: Construction of a fusion enzyme for astaxanthin formation and its characterisation in microbial and plant hosts: A new tool for engineering ketocarotenoids
Source: Metab Eng. 2019 Mar;52:243–52. doi: 10.1016/j.ymben.2018.12.006 (PMC6374281; doi:10.1016/j.ymben.2018.12.006)
Supplement: Supplementary file 10 — Supplementary material [file mmc5.pptx]

## Slide 1
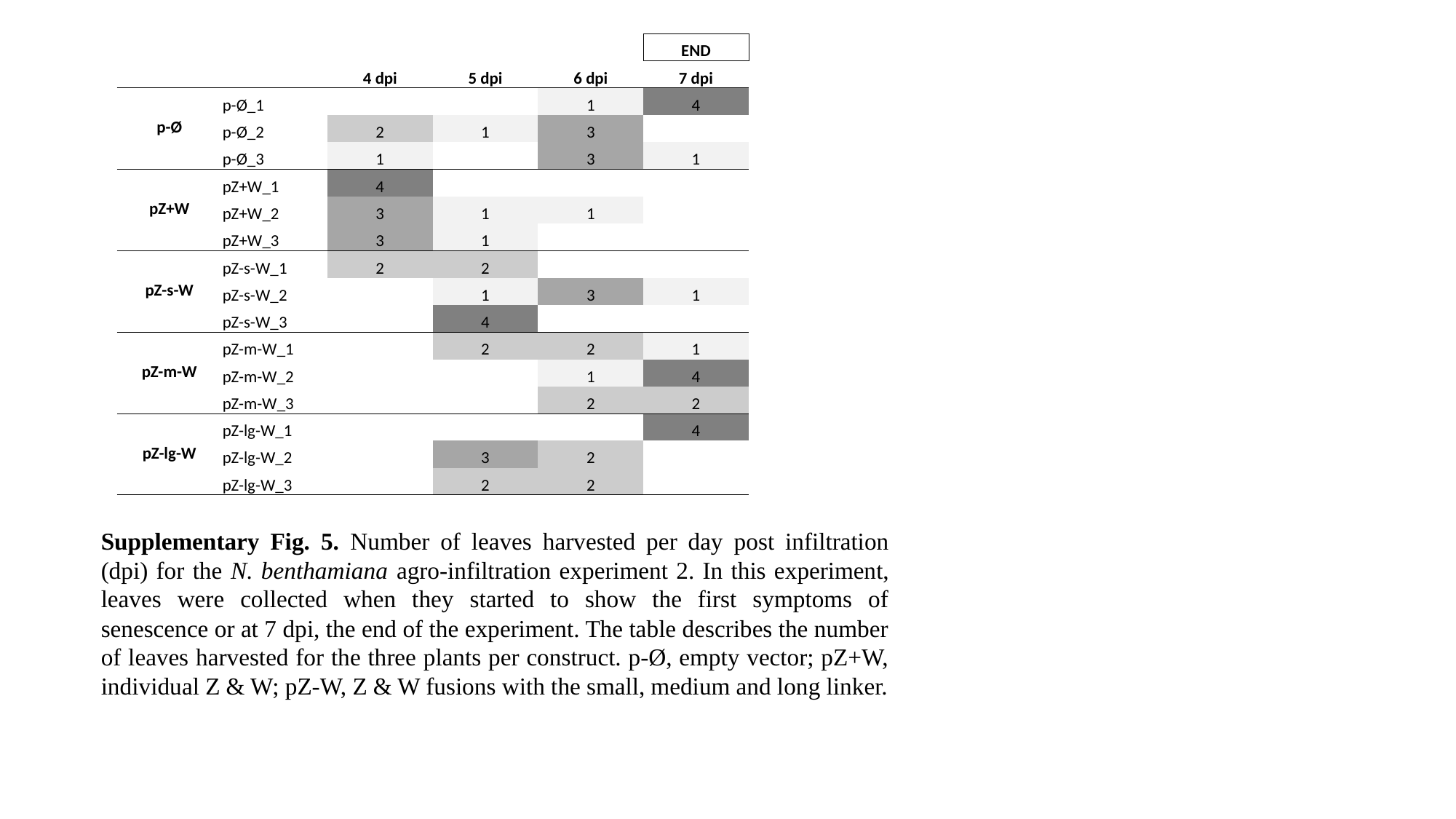

| | | | | | END |
| --- | --- | --- | --- | --- | --- |
| | | 4 dpi | 5 dpi | 6 dpi | 7 dpi |
| p-Ø | p-Ø\_1 | | | 1 | 4 |
| | p-Ø\_2 | 2 | 1 | 3 | |
| | p-Ø\_3 | 1 | | 3 | 1 |
| pZ+W | pZ+W\_1 | 4 | | | |
| | pZ+W\_2 | 3 | 1 | 1 | |
| | pZ+W\_3 | 3 | 1 | | |
| pZ-s-W | pZ-s-W\_1 | 2 | 2 | | |
| | pZ-s-W\_2 | | 1 | 3 | 1 |
| | pZ-s-W\_3 | | 4 | | |
| pZ-m-W | pZ-m-W\_1 | | 2 | 2 | 1 |
| | pZ-m-W\_2 | | | 1 | 4 |
| | pZ-m-W\_3 | | | 2 | 2 |
| pZ-lg-W | pZ-lg-W\_1 | | | | 4 |
| | pZ-lg-W\_2 | | 3 | 2 | |
| | pZ-lg-W\_3 | | 2 | 2 | |
Supplementary Fig. 5. Number of leaves harvested per day post infiltration (dpi) for the N. benthamiana agro-infiltration experiment 2. In this experiment, leaves were collected when they started to show the first symptoms of senescence or at 7 dpi, the end of the experiment. The table describes the number of leaves harvested for the three plants per construct. p-Ø, empty vector; pZ+W, individual Z & W; pZ-W, Z & W fusions with the small, medium and long linker.
