# Supplementary material for: Construction of a fusion enzyme for astaxanthin formation and its characterisation in microbial and plant hosts: A new tool for engineering ketocarotenoids
Source: Metab Eng. 2019 Mar;52:243–52. doi: 10.1016/j.ymben.2018.12.006 (PMC6374281; doi:10.1016/j.ymben.2018.12.006)
Supplement: Supplementary file 11 — Supplementary material [file mmc6.pptx]

## Slide 1
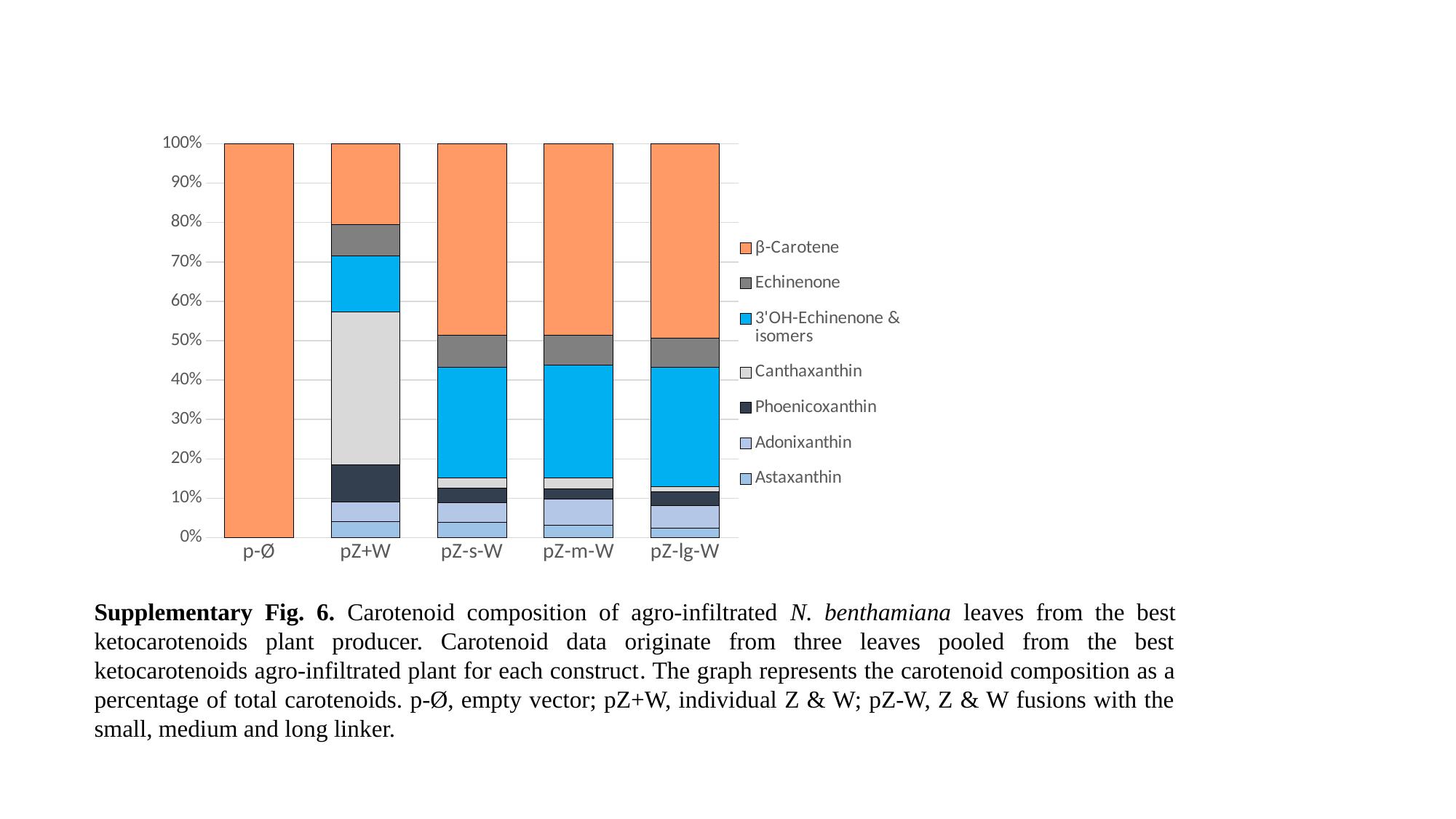

### Chart
| Category | Astaxanthin | Adonixanthin | Phoenicoxanthin | Canthaxanthin | 3'OH-Echinenone & isomers | Echinenone | β-Carotene |
|---|---|---|---|---|---|---|---|
| p-Ø | 0.0 | 0.0 | 0.0 | 0.0 | 0.0 | 0.0 | 481.3918418835472 |
| pZ+W | 17.728905246014563 | 21.226527130120758 | 41.336636827124195 | 167.49015835060075 | 61.561446159590886 | 34.27268617987658 | 88.82800676138224 |
| pZ-s-W | 14.197091629880417 | 18.112233159637302 | 12.933827486783402 | 9.69860571457577 | 101.09791681911942 | 29.18321658817804 | 175.82551196373572 |
| pZ-m-W | 11.594006750192833 | 25.216608245755356 | 9.82294700490141 | 9.939527687965155 | 107.98731959021318 | 28.237105519262702 | 182.40656246371637 |
| pZ-lg-W | 6.358107570664956 | 14.560460035219078 | 9.080237447509438 | 3.484082205620844 | 78.71211211880734 | 18.871411688317455 | 127.88918034318097 |Supplementary Fig. 6. Carotenoid composition of agro-infiltrated N. benthamiana leaves from the best ketocarotenoids plant producer. Carotenoid data originate from three leaves pooled from the best ketocarotenoids agro-infiltrated plant for each construct. The graph represents the carotenoid composition as a percentage of total carotenoids. p-Ø, empty vector; pZ+W, individual Z & W; pZ-W, Z & W fusions with the small, medium and long linker.
