# Supplementary material for: Construction of a fusion enzyme for astaxanthin formation and its characterisation in microbial and plant hosts: A new tool for engineering ketocarotenoids
Source: Metab Eng. 2019 Mar;52:243–52. doi: 10.1016/j.ymben.2018.12.006 (PMC6374281; doi:10.1016/j.ymben.2018.12.006)
Supplement: Supplementary file 12 — Supplementary material [file mmc7.pptx]

## Slide 1
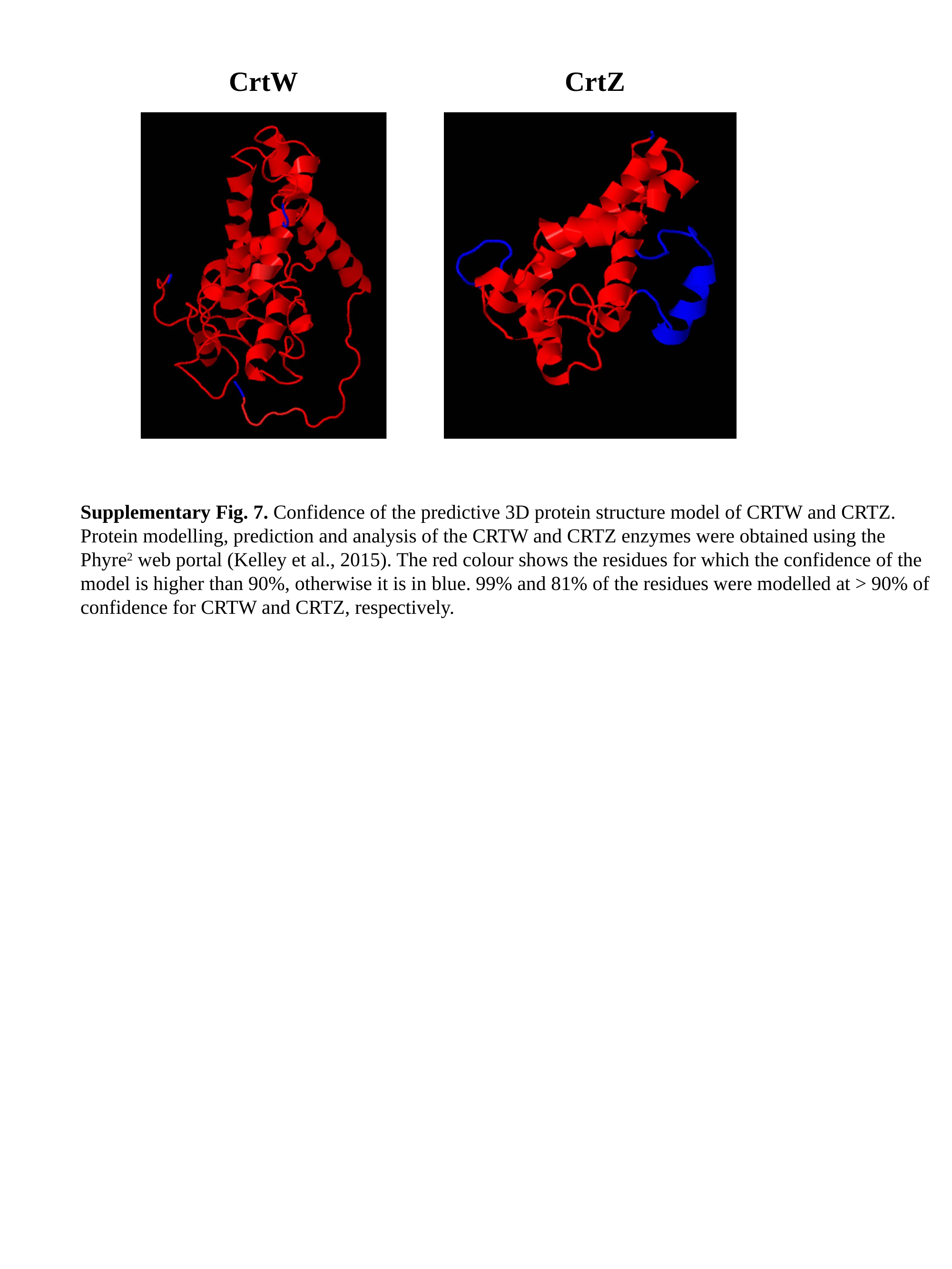

CrtW
CrtZ
Supplementary Fig. 7. Confidence of the predictive 3D protein structure model of CRTW and CRTZ. Protein modelling, prediction and analysis of the CRTW and CRTZ enzymes were obtained using the Phyre2 web portal (Kelley et al., 2015). The red colour shows the residues for which the confidence of the model is higher than 90%, otherwise it is in blue. 99% and 81% of the residues were modelled at > 90% of confidence for CRTW and CRTZ, respectively.
