# Supplementary material for: Construction of a fusion enzyme for astaxanthin formation and its characterisation in microbial and plant hosts: A new tool for engineering ketocarotenoids
Source: Metab Eng. 2019 Mar;52:243–52. doi: 10.1016/j.ymben.2018.12.006 (PMC6374281; doi:10.1016/j.ymben.2018.12.006)
Supplement: Supplementary file 13 — Supplementary material [file mmc8.pptx]

## Slide 1
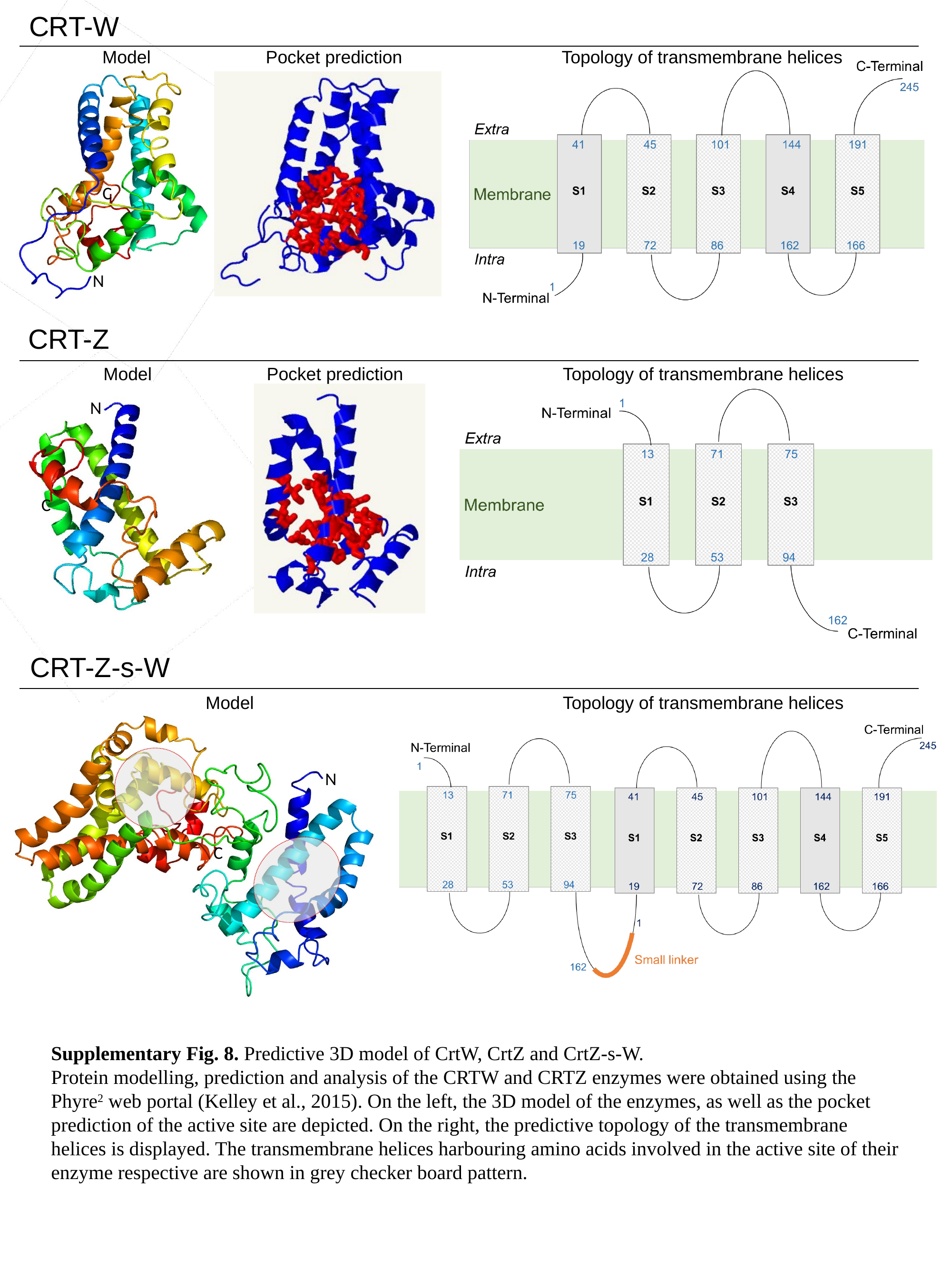

CRT-W
Model
Pocket prediction
Topology of transmembrane helices
C
N
CRT-Z
Model
Pocket prediction
Topology of transmembrane helices
N
C
CRT-Z-s-W
Model
Topology of transmembrane helices
N
C
Supplementary Fig. 8. Predictive 3D model of CrtW, CrtZ and CrtZ-s-W.
Protein modelling, prediction and analysis of the CRTW and CRTZ enzymes were obtained using the Phyre2 web portal (Kelley et al., 2015). On the left, the 3D model of the enzymes, as well as the pocket prediction of the active site are depicted. On the right, the predictive topology of the transmembrane helices is displayed. The transmembrane helices harbouring amino acids involved in the active site of their enzyme respective are shown in grey checker board pattern.
