# Supplementary material for: Construction of a fusion enzyme for astaxanthin formation and its characterisation in microbial and plant hosts: A new tool for engineering ketocarotenoids
Source: Metab Eng. 2019 Mar;52:243–52. doi: 10.1016/j.ymben.2018.12.006 (PMC6374281; doi:10.1016/j.ymben.2018.12.006)
Supplement: Supplementary file 14 — Supplementary material [file mmc9.pptx]

## Slide 1
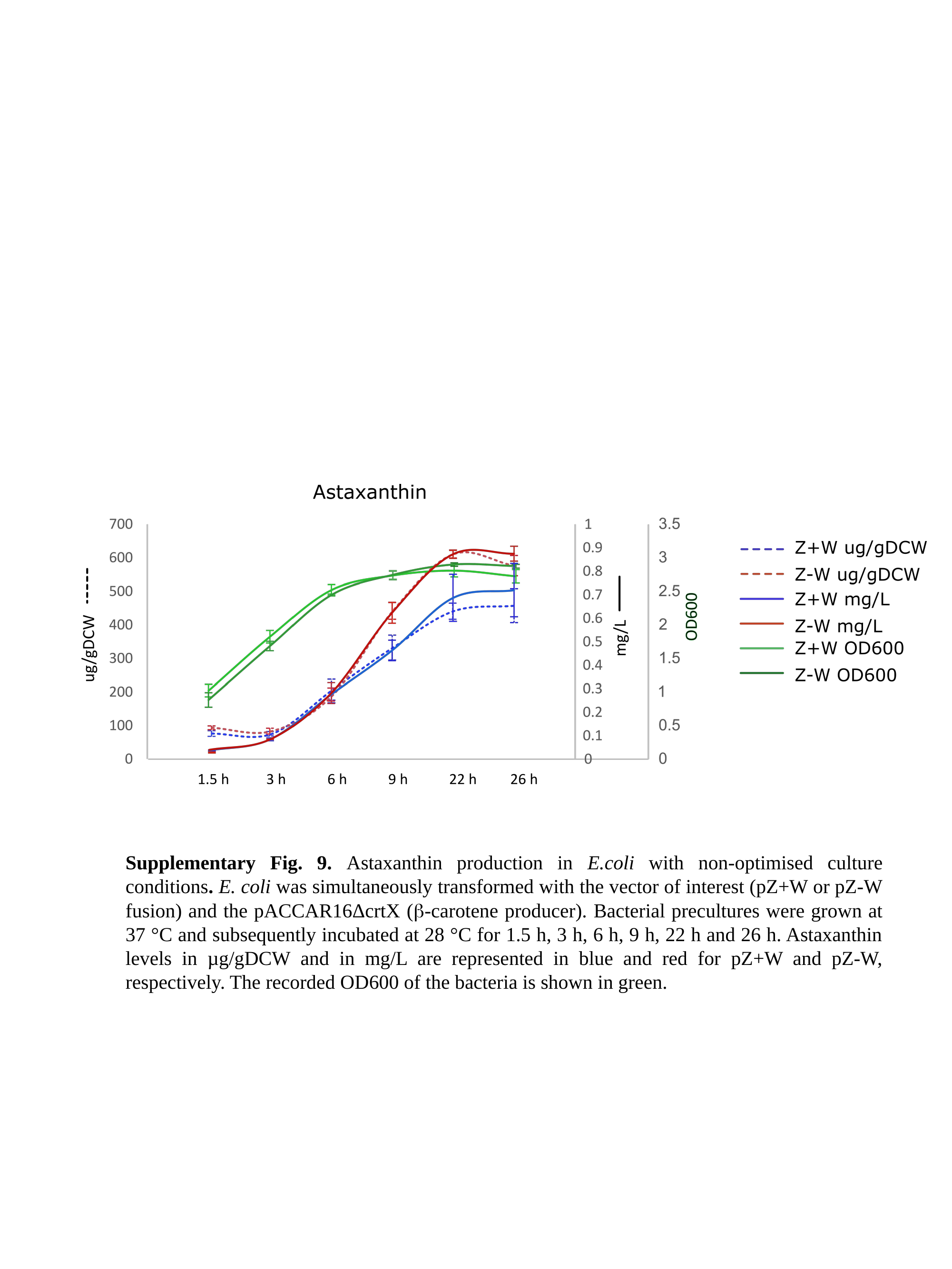

Supplementary Fig. 9. Astaxanthin production in E.coli with non-optimised culture conditions. E. coli was simultaneously transformed with the vector of interest (pZ+W or pZ-W fusion) and the pACCAR16ΔcrtX (β-carotene producer). Bacterial precultures were grown at 37 °C and subsequently incubated at 28 °C for 1.5 h, 3 h, 6 h, 9 h, 22 h and 26 h. Astaxanthin levels in µg/gDCW and in mg/L are represented in blue and red for pZ+W and pZ-W, respectively. The recorded OD600 of the bacteria is shown in green.
